# Positive Selection Drives Mitochondrial Gene Rearrangement in Sternorrhyncha (Insecta: Hemiptera)

**DOI:** 10.1002/ece3.71789

**Published:** 2025-07-20

**Authors:** Tian‐You Zhao, Jiu‐Feng Wei, Cai‐Feng Li, Ya‐Nan Chen, Liang Lü, Fang Wang

**Affiliations:** ^1^ Ministry of Education Key Laboratory of Molecular and Cellular Biology, Hebei Collaborative Innovation Center for Eco‐Environment, Hebei Key Laboratory of Animal Physiology, Biochemistry and Molecular Biology, College of Life Sciences Hebei Normal University Shijiazhuang Hebei China; ^2^ Department of Entomology and MOA Key Lab of Pest Monitoring and Green Management College of Plant Protection China Agricultural University Beijing China; ^3^ College of Plant Protection Shanxi Agricultural University Jinzhong Shanxi China

**Keywords:** evolutionary rate, gene rearrangement, mitochondrial genome, selection pressure, Sternorrhyncha

## Abstract

Sternorrhyncha, a suborder of Hemiptera, comprises sap‐feeding insects with piercing‐sucking mouthparts, most of which are important agricultural and forestry pests, including aphids, psyllids, whiteflies, and scale insects. While the mitochondrial genome is a highly accessible molecular source for high‐level and large‐scale phylogenetic studies, a comprehensive mitochondrial phylogenomic investigation of Sternorrhyncha has been lacking. This deficiency is primarily attributable to the challenges associated with obtaining mitochondrial genomes from Coccoidea. We have constructed the largest mitochondrial dataset for Sternorrhyncha to establish phylogenetic relationships, to examine the interrelationships, and to assess the phylogenetic results. Based on phylogenetic trees and mitogenomic gene arrangement synapomorphies, our findings confirm a sister‐group relationship between Coccoidea and Aphidoidea, and demonstrate the affiliation of Aclerdidae with Coccidae. Additionally, we have examined the mitochondrial gene rearrangements in Sternorrhyncha. Mitochondrial genes in Coccoidea and Aleyrodoidea display a notable prevalence of translocation and a high proportion of positive selection pressures, which correlates with their large species diversity. Conversely, most aphid genes are under negative selection pressure, and pairwise identity analysis reveals relatively modest low variation among aphid lineages, highlighting a paradox of species diversification underlain by conserved mitochondrial genomic changes.

## Graphical abstract

We have constructed the largest mitochondrial dataset for Sternorrhyncha to establish phylogenetic relationships. The mitochondrial genes of Coccoidea and Aleyrodoidea exhibit a notable prevalence of translocation and a high proportion of positive selection pressures. In Sternorrhyncha, the region next to the *CYTB* and *ND1*‐*rrnL*‐*rrnS* gene blocks represents a hotspot for gene translocation events. In our results, aphids have undergone a rapid species diversification decoupled from genetic evolution, leading to a lopsided in species diversity relative to mitochondrial conservativeness.

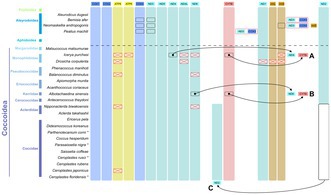

## 1 Introduction

Sternorrhyncha is accepted as a suborder of Hemiptera, including four main groups, Aphidoidea (aphids), Coccoidea (scale insects), Aleyrodoidea (whiteflies), and Psylloidea (psyllids), with approximately 20,000 described species worldwide (Cho et al. 2019; Cook et al. 2002; García Morales et al. 2016; Johnson et al. 2018). Sternorrhynchans are characterized by distinctive piercing‐sucking mouthparts and exhibit complex life cycles encompassing multiple hosts, parthenogenesis, polymorphism, and gall formation, all of which significantly contribute to their evolutionary radiations (Goodchild 1966; Gullan and Martin 2009; Song et al. 2016). Many sternorrhynchan species serve as economically important pests, inflicting millions of dollars in damage to fruit crops and ornamental plants due to their direct feeding damage, rapid reproductive rates, and capacity to transmit plant diseases and pathogens, such as *Schizaphis graminum* (aphid) (Royer et al. 2015), *Parasaissetia nigra* (scale insect) (Lin et al. 2017), and *Bemisia tabaci* (whitefly) (Bertin et al. 2021; Polston et al. 2014). Sternorrhyncha is of considerable interest to entomologists, largely attributable to its profound biological implications (Oberemok et al. 2023).

Sternorrhyncha is universally accepted as a monophyletic group, sister to all other hemipterans (Euhemiptera), which was strongly supported by both morphological and molecular studies (Cui et al. 2013; Johnson et al. 2018; Li et al. 2017; Song et al. 2019). However, the phylogenetic relationship of the four superfamilies within Sternorrhyncha is still debated. A hypothetical relationship of (Aphidoidea + (Coccoidea + (Psylloidea + Aleyrodoidea))), proposed based on the morphology of the alimentary canal (Goodchild 1966), has not been revisited or corroborated any longer. Some studies using morphological characteristics, fossil evidence, mitogenomic data, and genomic data have substantiated the classification within Sternorrhyncha, which resolves into two sister groups: (1) Psylloidea + Aleyrodoidea, and (2) Aphidoidea + Coccoidea (Boulard 1988; Drohojowska et al. 2020; Liu et al. 2024; Lu et al. 2023; Xu et al. 2023). Moreover, some mitochondrial data suggest the hypothesis that Psylloidea is the sister group to the remainder of Sternorrhyncha, with the proposed phylogenetic relationship of (Psylloidea + (Aleyrodoidea + (Aphidoidea + Coccoidea))) (Li et al. 2017; Song et al. 2019). While recent phylogenomic studies suggest that Aleyrodoidea is the earliest branching lineage within Sternorrhyncha, with a proposed phylogenetic relationship of (Aleyrodoidea + (Psylloidea + (Aphidoidea + Coccoidea))) (Johnson et al. 2018; Kieran et al. 2019; Misof et al. 2014; Song et al. 2024). Except for the first hypothesis, which has not occurred in any subsequent studies, the latter three are generally occurring in current studies, with the sister relatedness of Aphidoidea and Coccoidea remains reasonably constant, but the placement and relationship of Psylloidea and Aleyrodoidea are subjects of contention and controversy.

Mitochondrial genomes are extensively employed in evolutionary and phylogenetic investigations, attributed to their stable gene composition, maternal inheritance, and a low recombination level (Kelava et al. 2021; Li et al. 2017; Nie et al. 2021). Furthermore, several previous studies have employed mitogenomes to examine the phylogenetic relationship within Sternorrhyncha (Cui et al. 2013; Li et al. 2017; Song et al. 2016, 2019). However, on one hand, the shortage of the mitogenomes of scale insects left a large space for improvement in resolving the internal relationship within Coccoidea. Mitogenomes of scale insects have been increasingly sequenced since 2019, with 17 coccid mitogenomes currently available (Hou et al. 2023). On the other hand, few studies have gone further and investigated the site and structural evolution of the mitochondrial genome in Sternorrhyncha, particularly the Coccoidea, whose mitochondrial genomes exhibit distinct characteristics in terms of their unique gene order (Liu et al. 2020).

Scale insects are major agricultural and forestry pests. However, mitogenome data remain scarce for most known species, with only a minute fraction represented in public databases, thus sequencing is urgently needed to expand genomic resources for scale insects. This study seeks to explore the evolution of sternorrhynchan mitochondria from multiple angles, including a summary of the gene order patterns, an examination of selective pressure variation, and an analysis of mean pairwise identity among the major clades. In addition, we evaluate the reliability of mitochondrial phylogenetic relationships by various metrics and assess the phylogenetic signals at key nodes using likelihood mapping. Our study provides a unique insight into the mitochondrial evolution in Sternorrhyncha, which is valuable for future research on Hemiptera evolution.

## 2 Materials and Methods

### 2.1 Taxon Sampling and DNA Extracting

*Parthenolecanium corni* was collected from *Robinia pseudoacacia* in Huludao (40°42′ N, 120°56′ E), Liaoning Province of China on July 24 of 2019. *Ceroplastes floridensis*, *C. rusci*, and *Parasaissetia nigra* were collected respectively from Haikou (20°2′ N, 110°20′ E; July 28, 2020), Wenchang (19°32′ N, 110°47′ E; July 29, 2021), and Wuzhishan (18°46′ N, 109°31′ E; August 7, 2020) in Hainan Province of China. The corresponding host plants were *Thalia dealbata* , *Cocos nucifera* , and *Hibiscus rosasinensis* , respectively. These four species are all classified within Coccidae.

Total DNA was extracted from each species using QIAamp DNA Mini Kit (Qiagen, Germany) according to the extraction protocol. The genomic DNA library was generated, yielding 10 Gb of clean data by the Pair‐End 150 sequencing method on the Illumina HiSeq 6000 platform.

### 2.2 Mitogenome Assembly and Annotation

The complete mitochondrial genomes were iteratively obtained by GetOrganelle v1.7.2a (Jin et al. 2020). The protein‐coding genes (PCGs), rRNAs, and tRNAs of all mitochondrial genes were uniformly annotated using MitoZ v3.3 (Meng et al. 2019). The codon adaptation index (CAI) and relative synonymous codon usage (RSCU) were calculated by the CAI module (Lee 2018). A graphical map of the annotated circular mitogenome was produced via the OGDRAW tool 1.3.1 (Greiner et al. 2019).

### 2.3 Sequence Alignment and Dataset Selection

The amino acid sequences of PCGs and two rRNA genes were aligned using the default strategy in MAFFT v7.310 (Katoh and Standley 2013). The nucleotide sequences of PCGs were aligned using TranslatorX v1.1 (Abascal et al. 2010), according to the aligned amino acid sequence in the previous step. We compiled three datasets for further phylogenetic analyses. The alignments of the three datasets were derived from the output data of TranslatorX. Ambiguous sites and misaligned positions from the three datasets were trimmed using ClipKIT v2.3.0 (Steenwyk Iii et al. 2020) with smart‐gap mode. The aligned and trimmed sequences were concatenated into a matrix by PhyloSuite v1.2.3 (Zhang et al. 2020). Finally, the first dataset was composed of complete nucleotide sequences of 13 PCGs and two rRNA gene sequences (P123R dataset; 14,402 bp). The second dataset comprised nucleotide sequences of two rRNA genes and 13 PCGs without the third site (P12R dataset; 10,530 bp). The amino acid sequences of 13 PCGs with the termination codon excluded constitute the third dataset as AA dataset (3,872 AA).

### 2.4 Reconstruction of Phylogeny

The ingroup taxa included all representatives sampled from Aleyrodoidea, Psylloidea, Aphidoidea, and Coccoidea (Table S1). The outgroup taxa included those from Auchenorrhyncha, Coleorrhyncha, and Heteroptera (Hemiptera). *Thrips palmi* (Thysanoptera, Thripoidea) was used to root the resulting trees. Phylogenetic analyses were performed based on the three datasets using maximum likelihood (ML).

The ML inference was conducted in IQTREE v2.1.2 (Minh et al. 2020). Substitution models were compared and selected according to the Bayesian Information Criterion (BIC) using ModelFinder (Kalyaanamoorthy et al. 2017). Three partition schemes were applied to each matrix: (1) no partition (NP); (2) full partition (FP), which provides the best‐fitting model for each individual gene; and (3) merged partition (MP), which implements a greedy strategy that begins with the full partition model and subsequently merges gene pairs until no further improvement in model fit is achieved. An edge‐unlinked model was specified for both FP and MP schemes. Furthermore, the “AA” matrix was used to build phylogenetic trees under the mixture model (mtInv+F + C60) (Le et al. 2008). The best‐score ML tree was generated by 4 independent runs and selected according to BIC, with nodal supports evaluated via 1000 ultrafast bootstraps (Hoang et al. 2018).

The metrics of saturation and signal‐to‐noise ratio (SNR) were computed using the Phylogenomics Toolkit v1.20.0 (Steenwyk et al. 2021). A value of *R* ^2^ approaching 1 indicates a reduced saturation of the dataset. A higher SNR indicates a reduced likelihood of compositional bias impacting the dataset. The phylogenetic trees were visualized using iTOL v 6.8.1 (Letunic and Bork 2021).

### 2.5 Hypothesis Testing for Topologies

The quartet likelihood mapping in IQTREE v2.1.2 was used to assess the informative resolution of three datasets, effectively visualizing the tree‐likeness of all quartets in a single graph, thus providing a robust interpretation of the phylogenetic content of a dataset. The Four‐cluster Likelihood Mapping (FcLM) was used to evaluate key nodes regarding Sternorrhyncha.

For all FcLM analyses, the following topologies and groups were defined:

T1: (G1, G2)–(G3, G4); T2: (G1, G3)–(G2, G4); T3: (G1, G4)–(G2, G3).

Hypothesis 1 *Sister group relationship between Coccoidea and Aphidoidea*.

Groups: G1: Psylloidea; G2: Aleyrodoidea; G3: Coccoidea; G4: Aphidoidea.

Hypothesis 2 *The relationship between* Didesmococcus koreanus *and Aclerdidae*.

Groups: G1: Aclerdidae; G2: *Ericerus pela*; G3: *Didesmococcus koreanus*; G4: others of Coccoidea.

### 2.6 Assessment of Selective Pressures and Mean Pairwise Identity

To assess the selective pressures on the mitogenomes of different lineages across Sternorrhyncha, 131 species containing all 13 PCGs were retained for analysis; those marked with a caret (^) were excluded (Table S1). The ratio of nonsynonymous (dN) to synonymous (dS) substitution rates was used to infer purifying selection (dN/dS < 1) and positive selection (dN/dS > 1) (Hurst 2002; Mugal et al. 2014; Peterson and Masel 2009). Selection pressure at each site within genes was assessed using the Single‐Likelihood Ancestor Counting (SLAC) method, which integrates maximum likelihood (ML) and counting methods to deduce the dN/dS ratio on a per‐site basis for a given coding alignment and corresponding phylogeny (Kosakovsky Pond and Frost 2005). A site is deemed to be under positive selection pressure if the *p* value for positive selection < 0.05. Conversely, the site is categorized as under negative selection pressure if the *p* value of negative selection < 0.05. Sites with a *p* value ≥ 0.05 are considered to lack significant selection pressure and are therefore included in the “other” category.

The adaptive Branch‐Site Random Effects Likelihood (aBSREL), allowing the variation of selective pressures among both codon sites and individual branches in the phylogeny (Smith et al. 2015), was used to determine the optimal number of dN/dS classes for each branch lineage, with each branch node typically comprising two or three categories (dN/dS > 1, dN/dS < 1, dN/dS = 1). The distribution of locations within the concatenated mitochondrial genes has been quantified for each category. The above two methods, SLAC and aBSREL, with a topology derived from the best tree of the AA dataset, were all implemented in Hyphy v2.5.62 (Kosakovsky Pond et al. 2020). The Hyphy visualization website (http://vision.hyphy.org) was used to examine the proportion under selection pressure sites.

Pairwise identity refers to the number of identical sites (including gaps) between two aligned sequences divided by the alignment length, which serves as an indicator of the evolutionary rate of sequences (Chen, Liang, and Zhang 2017). Values range from 0 to 1, where 0 signifies slow evolution and a lack of diversity at the specified site, while 1 denotes rapid evolution with each character appearing only once. In our analysis, mean pairwise identities were employed to assess the degree of sequence variation within a clade. We used the Phylogenomics Toolkit for mean pairwise identities analysis, performing calculations on a dataset of 13 PCGs and 2 rRNAs.

## 3 Results

### 3.1 Mitochondrial Genome and Protein‐Coding Genes

**FIGURE 1:**
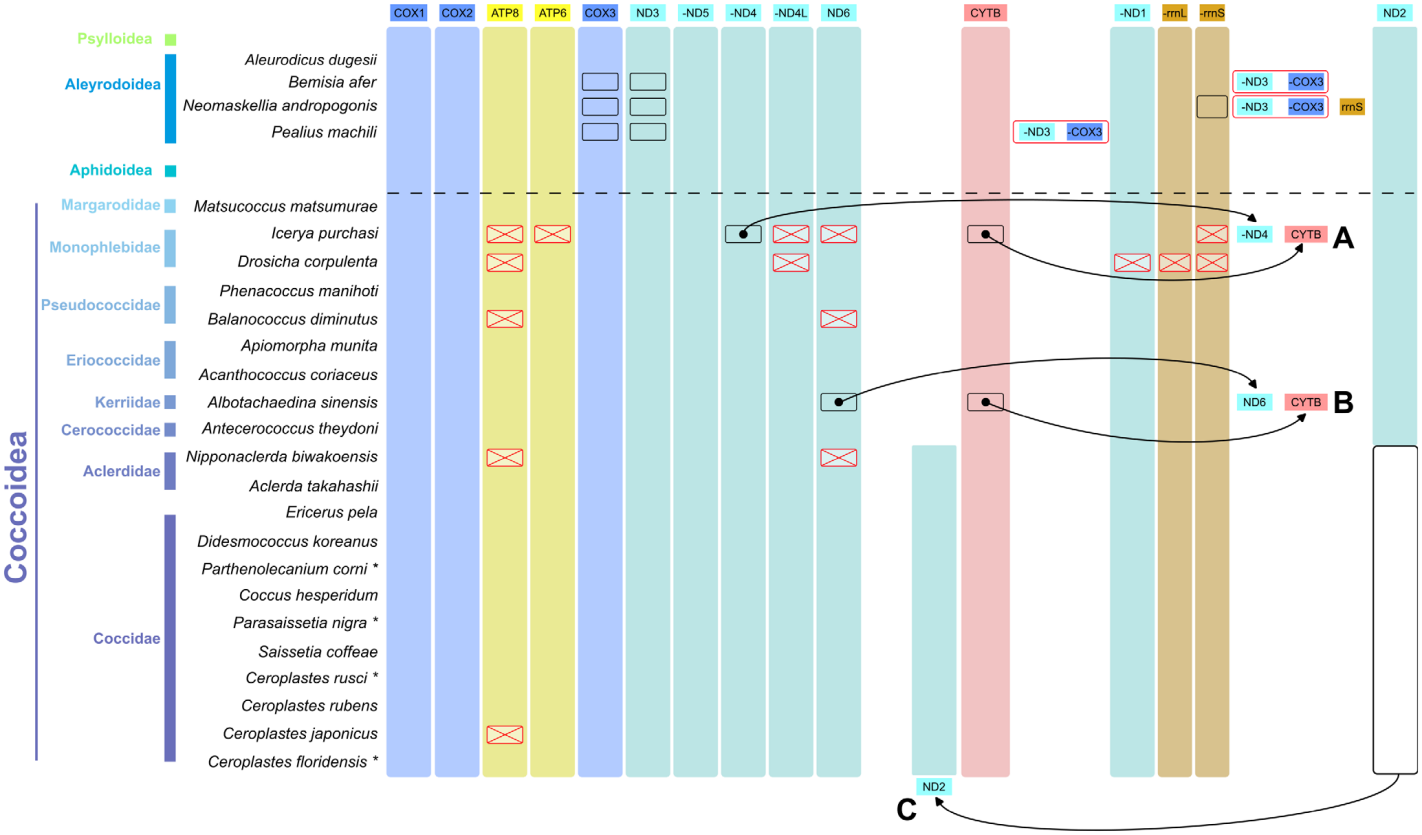
Comparison of mitochondrial genome arrangements of Coccoidea. Genes transcribed from the majority strand are oriented from left to right, while those from the minority strand are prefixed with a “‐” symbol to denote their reverse transcription direction. The red box indicates the gene that is missing in the sample. (A) In *Icerya purchasi* , the *ND4* and *CYTB* have moved; (B) In *Albotachaedina sinensis*, the *ND6* and *CYTB* have moved; C: Within the families Aclerdidae and Coccidae, the *ND2* has changed its position across all species.

Complete mitogenomes of *Ceroplastes floridensis* (15,086 bp), *C. rusci* (14,952 bp), *Parasaissetia nigra* (15,632 bp), and *Parthenolecanium corni* (15,135 bp) were sequenced and annotated. The mitogenomes exhibit the same gene arrangement for the 13 PCGs, with 22 genes located on the forward strand and others on the reverse strand in *C. floridensis and Parasaissetia nigra* (Figure S1 and Tables S2–S5). However, the tRNA‐Cys in *C. rusci* and the tRNA‐Ala in *Parthenolecanium corni* are located on the forward strand. These species maintain the most common gene order (excluding tRNA) of Coccidae inside the gene rearrangement region of Coccoidea (Figure 1).

Of the 13 PCGs, *ATP8* is the shortest at 141 bp throughout the four species sequenced in the current study, while *ND5* is the longest in *C. floridensis* (1,599 bp) and *Parasaissetia nigra* (1,600 bp), and *COX1* is the longest gene in *C. rusci* (1,527 bp) and *Parthenolecanium corni* (1,530 bp). In terms of AT content among the 13 PCGs, *ND6* has the highest AT content in *C. rusci* and *Parasaissetia nigra* , while *ND4L* displays the highest AT content in *C. floridensis* and *Parthenolecanium corni* (Table S6). The RSCU analysis across the four species suggests that a total of 64 codons were utilized (Figure S2). All 13 PCGs use ATN as the start codon. Notably, the codon ATA (Met) was identified as the most prevalent codon in PCGs, occurring over 1000 times across the four species (see Tables S7–S10).

### 3.2 Phylogenetic Relationships of Sternorrhyncha and the Hypothesis of Conflict Nodes

A total of 10 trees were established based on the three datasets (AA, P12R, and P123R) with three partition schemes, and the C60 mixture model exclusively for the AA matrix (Figures [Link], [Link], [Link], [Link], [Link], [Link], [Link], [Link], [Link], [Link]). The information including AIC, BIC, saturation, SNR, and bootstrap of each node is summarized in Table S11. According to the BIC, the trees built using the MP scheme were determined to be the best for the AA and P12R datasets, whereas the FP scheme was best for the P123R dataset. The AA dataset with the MP scheme had the highest level of saturation. Regarding SNR, the AA dataset had the highest SNR compared with the other datasets. Among the best trees for each dataset, the P123R dataset had the greatest quantity of dependable nodes (BS≥ 95).

The phylogenetic relationships of Sternorrhyncha have been consistently demonstrated throughout the best trees of each dataset. All analyses strongly supported the monophyly of Sternorrhyncha, with Psylloidea occupying the most basal position, sister to the remainder of this suborder. Aleyrodoidea is the sister to the clade comprising the sibling superfamilies, Coccoidea and Aphidoidea.

**FIGURE 2:**
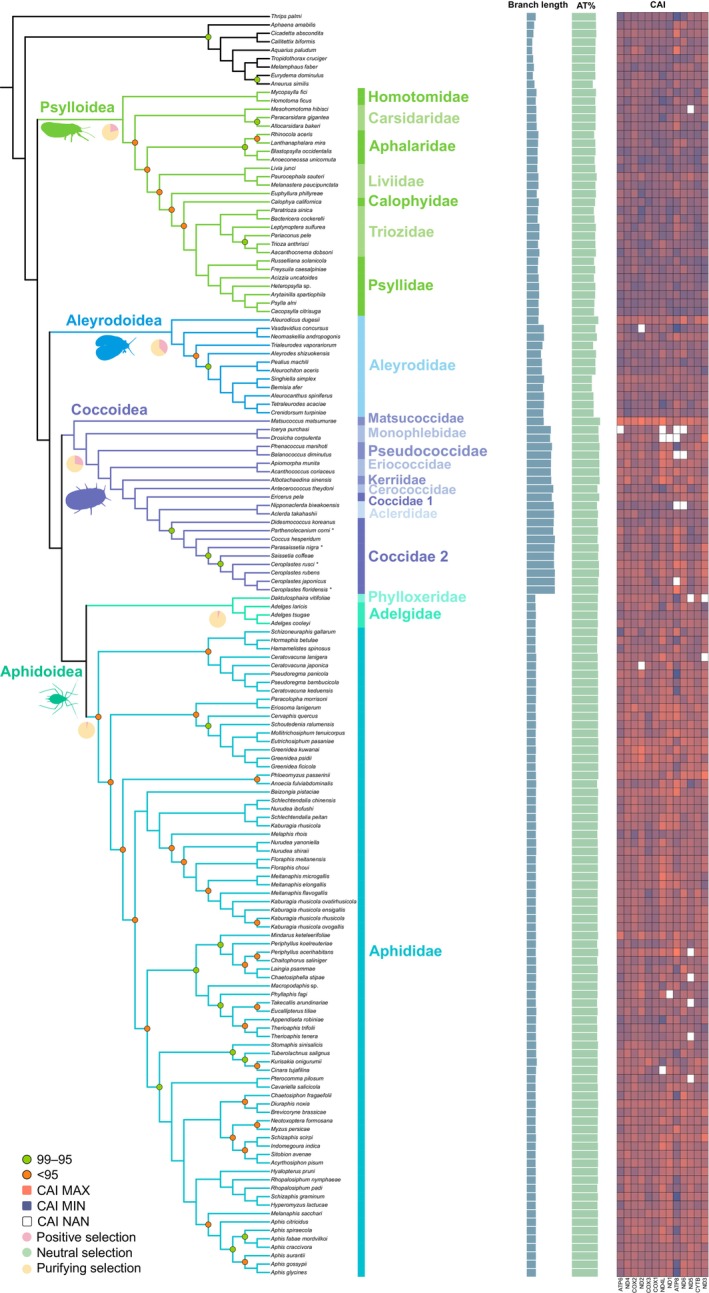
Phylogenetic relationships of Sternorrhyncha are presented based on optimal tree of AA dataset. The species followed by an asterisk (*) are sequenced in this study. Nodes with 100 bootstrap supports are not labeled. Nodes with support < 95 are labeled in orange, and those with support between 95 and 99 are labeled in green. The pie chart below the node represents the proportion of selected pressure sites based on the aBSREL results. The first column on the right side of the figure represents branch length; the second column shows the AT content of the 13 PCGs; the third column indicates the CAI (Codon Adaptation Index) of the 13 PCGs.

Concerning the internal relationships within Psylloidea, both the P123R and P12R matrices exhibit strong nodal support, although the AA dataset falls short of sufficient phylogenetic signal; yet, the topologies are consistent among all datasets (Figure 2). The internal familial relationships within Coccoidea exhibit consistent representations across all three datasets (Figure 2). Matsucoccidae and Monophlebidae occupy the most basal positions, whereas Aclerdidae is embedded inside Coccidae, resulting in a paraphyletic Coccidae. Consequently, the relationship between Aclerdidae and Coccidae, especially the position of *Ericerus pela*, needs further investigation. Within Aphidoidea, Phylloxeridae and Adelgidae are confirmed as sibling groups (forming the former “Phylloxeroidea”), together sister to the remaining aphidoids. The internal familial relationship of Aphididae exhibits inadequate phylogenetic signal in all examined matrices.

We proposed two hypotheses to investigate the key unreliable nodes in the best tree of each dataset. The results of likelihood mapping indicate that all three matrices of Hypothesis 1 strongly endorse the sister relationship between Coccoidea and Aphidoidea (Table 1). For Hypothesis 2, the P12R and P123R matrices demonstrate a closer relatedness between Aclerdidae and *D. koreanus* , which is also supported by the corresponding phylogenetic trees despite the uncertain results from the AA matrix (Table 1).

**TABLE 1:** Results of Four‐cluster Likelihood Mapping (FcLM) for the two hypotheses based on three datasets.

Hypothesis	Topologies and groups	P12R	P123R	AA
Hypothesis 1	T1:(Psylloidea, Aleyrodoidea)‐ (Coccoidea, Aphidoidea)	**99.93**	**99.86**	**99.41**
Hypothesis 1	T2:(Psylloidea, Coccoidea)‐ (Aleyrodoidea, Aphidoidea)	0.00	0.00	0.00
Hypothesis 1	T3:(Psylloidea, Aphidoidea)‐(Aleyrodoidea, Coccoidea)	0.03	0.06	0.34
Hypothesis 2	T1:(Aclerdidae, *Ericerus pela*)‐ (*Didesmococcus koreanus*, others of Coccoidea)	18.75	18.75	43.75
Hypothesis 2	T2:(Aclerdidae, *Didesmococcus koreanus*)‐(*Ericerus pela*, others of Coccoidea)	**62.50**	**56.25**	**56.25**
Hypothesis 2	T3:(Aclerdidae, others of Coccoidea)‐ (*Ericerus pela*, *Didesmococcus koreanus*)	6.25	12.50	0.00

*Note:* Bold values highlight the best‐supported topology based on maximum signal proportion. These values are not used to calculate statistically significant differences.

### 3.3 Rearrangement of Mitochondrial Genes

To understand the gene rearrangement of Sternorrhyncha, 13 PCGs and 2 rRNA genes of some representative species were selected for comparative analysis (Figure 1). In Aphidoidea and Psylloidea, the gene order is highly conserved, with no observable rearrangements. The mitochondrial genome rearrangements of Aleyrodoidea exhibit three distinct patterns. A common gene rearrangement, such as that observed in *Bemisia afer*, typically involves the insertion of the *ND3*‐*COX3* gene block between the *ND1*‐*rrnL*‐*rrnS* and *ND2* gene blocks. The second pattern resembles the gene configuration of *Neomaskellia andropogonis* and *Vasdavidius concursus*, where the *rrnS* is positioned between the *COX3* and *ND2*. In the case of *V. concursus*, the *rrnS* gene annotation is absent, possibly due to an incomplete assembly of the mitochondrial genome. Additionally, *Pealius machili* and *Aleurochiton aceris* demonstrate a unique rearrangement in which the *ND3*‐*COX3* gene block is interposed between the *CYTB* and *ND1*‐*rrnL*‐*rrnS* gene blocks. In scale insects, mitochondrial gene arrangements are categorized into three primary types. The placement of *ND2* in Aclerdidae and Coccidae has shifted between *ND6* and *CYTB*, potentially indicating a characteristic feature of both families. The incorporation of the *ND4*‐*CYTB* and *ND6*‐*CYTB* gene blocks is corroborated by evidence from *Icerya purchasi* and *Albotachaedina sinensis*, respectively. In the mitochondrial genome of Sternorrhyncha, the vicinity of the *CYTB* and *ND1*‐*rrnL*‐*rrnS* gene blocks is a hotspot for gene translocation events.

### 3.4 Selective Pressures and Mean Pairwise Identity of Sternorrhyncha

**FIGURE 3:**
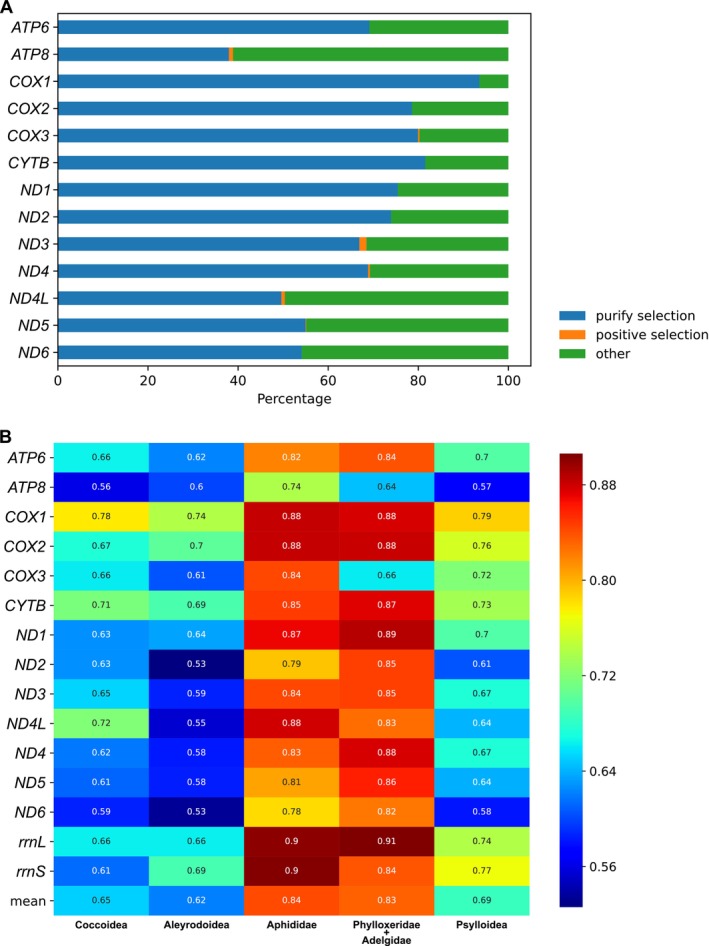
Selection pressures on sites of the 13 PCGs and mean pairwise identity for the five major superfamilies within Sternorrhyncha. (A) Distribution of selection pressures on sites as shown by a histogram. Sites under purifying selection are blue, those under positive selection are orange, and sites classified as “other” are green. (B) The mean pairwise identity represents the evolutionary rate. The higher values indicate more conservation (red), while lower values reflect greater divergence (blue).

Among the examined genes, *COX1* exhibits the highest proportion of purifying selection, indicating strong evolutionary conservation (Figure 3A). In contrast, *ATP8* shows a lower selective pressure and the lowest proportion of purifying selection. *ATP8, COX3, ND3, ND4, ND4L*, and *ND5* contain sites under positive selection, highlighting particular regions of adaptive evolution. aBSREL analysis of selection pressure on concatenated mitochondrial genes across different branches shows that no sites underwent neutral selection throughout the major clades (Figure 2). Moreover, positive selection was detected at 21.00% of sites in Psylloidea, 37.41% in Aleyrodoidea, and 27.81% in Coccoidea. In contrast, only 3%–4% of sites in Aphidoidea exhibit indications of positive selection (Figure 2).

The mean pairwise identity across 15 genes in Sternorrhyncha is 0.72. Among the taxa, the Aphidoidea clade has a high mean pairwise identity of 15 genes, with values of 0.84 and 0.83, suggesting a reasonably conserved mitochondrial evolutionary rate in this lineage. Conversely, Aleyrodoidea has the lowest evolutionary rate at 0.62, while Coccoidea and Psylloidea have the rates of 0.65 and 0.69, respectively (Figure 3B). Among the sternorrhynchan genes examined, *ATP8* exhibits an average evolutionary rate of 0.62, making it the fastest evolving gene, while *COX1* has the highest average evolutionary rate of 0.81 and is identified as the slowest evolving gene.

## 4 Discussion

### 4.1 Mitochondrial Phylogeny of Sternorrhyncha

We gathered the mitochondrial genomic data for the greatest number of species within Sternorrhyncha, covering all major lineages of Sternorrhyncha to investigate the internal relationship of this suborder. All phylogenetic results from this study strongly confirm the monophyly of Sternorrhyncha, positioning it as the sister group to the remaining Hemiptera. The phylogenetic relationship of Sternorrhyncha is represented as Psylloidea + (Aleyrodoidea + (Coccoidea + Aphidoidea)). The sister relatedness between Coccoidea and Aphidoidea was strongly supported by nearly all prior morphological and molecular studies (e.g., Hou et al. 2023; Johnson et al. 2018; Liu et al. 2024; Lu et al. 2023; Misof et al. 2014; Song et al. 2024) and confirmed in our FcLM and phylogenetic analyses. The primary contention has been focused on which clade, Psylloidea or Aleyrodoidea, is the most basal. Pairwise identity detection suggests that Aleyrodoidea may represent one of the most ancient lineages within Sternorrhyncha. However, morphologically, psyllids, like other hemipteran insects, possess only a rudimentary filter chamber in their gut, attached with four malpighian tubules, which are thought to be an ancestral feature of hemipteran insects (Gullan and Martin 2009). While whiteflies, aphids, and scale insects all have a well‐developed filter chamber in the gut and have no or reduced malpighian tubules (Gullan and Martin 2009; Hodges and Evans 2005). Consequently, we hypothesized that Psylloidea may be the earliest lineage and serves as the sister group to all other sternorrhynchans. Studies based on transcriptome or genomic data predominantly endorsed Aleyrodoidea (Johnson et al. 2018; Misof et al. 2014; Song et al. 2024), while those using mitogenome data, aligning with our findings, supported Psylloidea (Hou et al. 2023; Song et al. 2019). We speculate that the outcome of this conflict may be influenced by the incongruent evolutionary history of nuclear and mitochondrial DNA. The phylogenetics based on ultraconserved elements (UCE) revealed a third pattern, indicating that the sibling Aleyrodoidea and Psylloidea together are sister to Coccoidea and Aphidoidea (Liu et al. 2024).

Coccoidea are generally divided into two informal groups, “archaeococcoids” and “neococcoids,” with the former regarded to bear more ancestral traits (Hodgson and Hardy 2013; Liu et al. 2024). Our study shows that Matsucoccidae and Monophlebidae are classified under the “archaeococcoids” and occupy the most basal positions, but they fail to form a monophyletic “archaeococcoids.” In addition, under the “neococcoids,” Aclerdidae is nested within the Coccidae clade, rendering the latter paraphyletic. In fact, the sister relationship of Aclerdidae and Coccidae has consistently been supported by morphological evidence (Hodgson and Hardy 2013; Vea and Grimaldi 2016). Similar outcomes have been achieved in previous studies using data from different data sources (Hou et al. 2023; Xu et al. 2023; Liu et al. 2024; Song et al. 2024). The synapomorphic features that distinguish these two families from other “neococcoids” include the presence of an anal cleft and anal plates, as well as the absence of octuform glands (Wang et al. 2019). However, our sampling for both Aclerdidae and Coccidae remains insufficient, so their relationship should be re‐examined once more taxa are sequenced.

Our findings categorize aphids into two sister groups, oviparous and ovoviviparous, in line with the prevailing classification (Jiang and Qiao 2011). Oviparous aphids encompass two families, Adelgidae and Phylloxeridae, which are considered to be more closely related to the ancient plan of aphids (Chen, Wang, et al. 2017; Heie and Wegierek 2009; Jiang and Qiao 2011). Ovoviviparous aphids, comprising more than 95% of all the aphidoid species, are commonly called true aphids, belonging to the family Aphididae (Chen, Wang, et al. 2017). Regrettably, the internal relationships within Aphididae remain unresolved due to the explosive radiations and the special diversification mechanisms, which have resulted in limited phylogenetic signal in the genetic data primarily used for phylogenetic analysis. Incorporating multiple data sources, such as endosymbionts, may help address these challenges (Donner et al. 2024; Jousselin et al. 2024; Ren et al. 2020).

### 4.2 Order Patterns of Mitochondrial Genes

The mitochondrial genome arrangement of most insects is relatively conserved, but certain groups may undergo rearrangements and changes over evolution (Boore 1999; Dowton et al. 2002). The analysis of rearrangements of mitochondrial tRNA genes is complicated due to inadequate sequence data and annotation discrepancies. Therefore, our study concentrates on 13 PCGs and 2 rRNA genes, revealing prominent and readily recognizable patterns of gene rearrangements in Sternorrhyncha. In Aphidoidea and Psylloidea, the gene order is highly conserved, with no observable rearrangements, consistent with previous studies (Chen, Wang, et al. 2017; Lu et al. 2023). The gene rearrangement in Aleyrodoidea primarily transpires at the *ND3*‐*COX3* locus, aligning with some previous studies (Ghosh et al. 2024; Lu et al. 2023). The mitogenomes of coccids display extensive gene rearrangement; however, the typical sternorrhynchan configuration is observed in most basal taxa, namely Matsucoccidae, Pseudococcidae, Eriococcidae, and Cerococcidae, despite the presence of some translocations in Monophlebidae and Kerriidae. These translocations can be regarded as stochastic occurrences caused by incorrect slipped‐strand mismatches or gene deletions after duplication (Dowton et al. 2003; Liu et al. 2023). The prominent change takes place in Coccidae and Aclerdidae. Both have an *ND2* positioned prior to *CYTB*, in contrast to the configuration found in other coccoid families, which again reinforces the affiliation of Aclerdidae with Coccidae. In comparison with more than 8500 species of scale insects, the existing mitogenomes are still very limited; hence, further evidence and observation of gene transfer are still necessary. In the mitochondrial gene rearrangements of Sternorrhyncha, the region next to the *CYTB* and *ND1*‐*rrnL*‐*rrnS* gene blocks represents a hotspot for gene translocation events.

### 4.3 Selective Pressure and Per‐Site Evolution

Mitochondrial genes demonstrate adaptive evolution in response to various selective pressures, and it is generally accepted that the evolution of mitogenomes is deterred by strong purifying selection (Castellana et al. 2011; Escalona et al. 2017; Liu et al. 2023). All involved genes in Sternorrhyncha, based on the gene selective pressure and mean pairwise identity, underwent purifying selection, with the *COX1* gene exhibiting strong evolutionary conservation and *ATP8* showing the highest evolutionary rate. These findings are consistent with some previous studies (Lu et al. 2023). Aleyrodoidea and Coccoidea have the highest fraction (≥ 25%) of positive selection pressured sites (Figure 2) and their mitogenomes have expressed all the categories of gene rearrangement present in Sternorrhyncha (Figure 1). The activeness in mitochondrial evolution is commensurate with the diversity of the two superfamilies (totally about 10,000 species), while the positive selection pressure appears to have induced mitochondrial rearrangements in these two clades, particularly in Coccoidea. Scale insects were found to require less energy due to their weak or inactive mobility, leading to adaptive evolution alongside their host organisms and promoting the accumulation of more nonsynonymous mutations in mitogenome PCGs (Lu et al. 2023). Despite the absence of observed mitochondrial gene rearrangement within Psylloidea to date, this group exhibits an elevated proportion of positively selected sites. We hypothesize that enhanced taxonomic sampling may facilitate the detection of mitochondrial gene rearrangement in psyllids.

Despite their considerable taxonomic diversity (exceeding 4000 species), genes within Aphidoidea exhibit high conservation and low divergence. Most of the aphid mitochondrial gene sites were primarily under purifying selection, while the positive selection pressure was comparatively minimal, indicating a tendency for gene conservation. Coupled with our phylogenetic findings, this indicates that aphids have experienced rapid species diversification decoupled from their mitochondrial genetic evolution, resulting in a disproportionately high species diversity relative to the mitochondrial conservativeness.

## 5 Conclusion

Our results clarified the internal relationships within this suborder from a mitochondrial perspective, demonstrating that Psylloidea represents the earliest diverging lineage and is the sister group to all other taxa of Sternorrhyncha. Additionally, our results reaffirm the close sister group relationship between coccids and aphids and the inclusion of “Aclerdidae” within Coccidae. The familial rank of “Aclerdidae” warrants further investigation utilizing additional data. While the three patterns of the major branching events of Sternorrhyncha exhibit a discernible dependency on data type, it is too precipitous to scapegoat the data property for the conflict topologies, as the preparation and substitution model used in phylogenetics have been found to significantly influence the results, particularly in the data‐intensive studies (Cai et al. 2020; Cai et al. 2022; Liu et al. 2019; Tihelka et al. 2020). The majority of nodes within the Coccoidea clade are well‐supported and exhibit minimal disagreement with previous studies. However, a notable limitation is the current deficiency in the taxonomic sampling within this group. Selecting appropriate data for the phylogenetics of Aphididae is a Gordian knot that may not be resolved in a short time. Therefore, continued research is essential to comprehensively clarify the phylogenetic relationships within Sternorrhyncha. We also conducted a comprehensive analysis of the mitochondrial genomic data of Sternorrhyncha, highlighting the distinctions among various taxa. Gene rearrangement analysis revealed a particular emphasis on Coccoidea and Aleyrodoidea, which exhibit the most pronounced mitochondrial evolution, both in terms of per‐site alteration and gene translocation. Most genes in aphids experienced negative selection pressure, and pairwise identity analysis revealed relatively modest variation among their branches, reflecting a more conserved evolutionary pattern. There is a critical need for more robust mitochondrial data on scale insects, as the real pattern of mitochondrial genomic evolution remains largely uncharacterized within this superfamily.

## Author Contributions

**Tian‐You Zhao:** software (equal), writing – original draft (lead), writing – review and editing (equal). **Jiu‐Feng Wei:** conceptualization (equal), supervision (equal), writing – review and editing (equal). **Cai‐Feng Li:** data curation (equal), supervision (equal), writing – review and editing (equal). **Ya‐Nan Chen:** investigation (equal), visualization (equal), writing – review and editing (equal). **Liang Lü:** conceptualization (equal), funding acquisition, project administration (equal), writing – review and editing (equal). **Fang Wang:** conceptualization (equal), data curation (equal), project administration (equal), writing – review and editing (equal).

## Conflicts of Interest

The authors declare no conflicts of interest.

## Supporting information


Figure S1.

Figure S2.

Figure S3.

Figure S4.

Figure S5.

Figure S6.

Figure S7.

Figure S8.

Figure S9.

Figure S10.

Figure S11.

Figure S12.

Tables S1–S11.

## Data Availability

Data to support this study are available from the National Center for Biotechnology Information (https://www.ncbi.nlm.nih.gov). The registration numbers are NC_067791 (*Ceroplastes floridensis*), PV269613 (*Ceroplastes rusci*), NC_067790 ( *Parasaissetia nigra* ), and PV269614 (*Parthenolecanium corni*).
