# Supplementary figures and images for: Positive Selection Drives Mitochondrial Gene Rearrangement in Sternorrhyncha (Insecta: Hemiptera)
Source: Ecol Evol. 2025 Jul 20;15(7):e71789. doi: 10.1002/ece3.71789 (PMC12277048; doi:10.1002/ece3.71789)

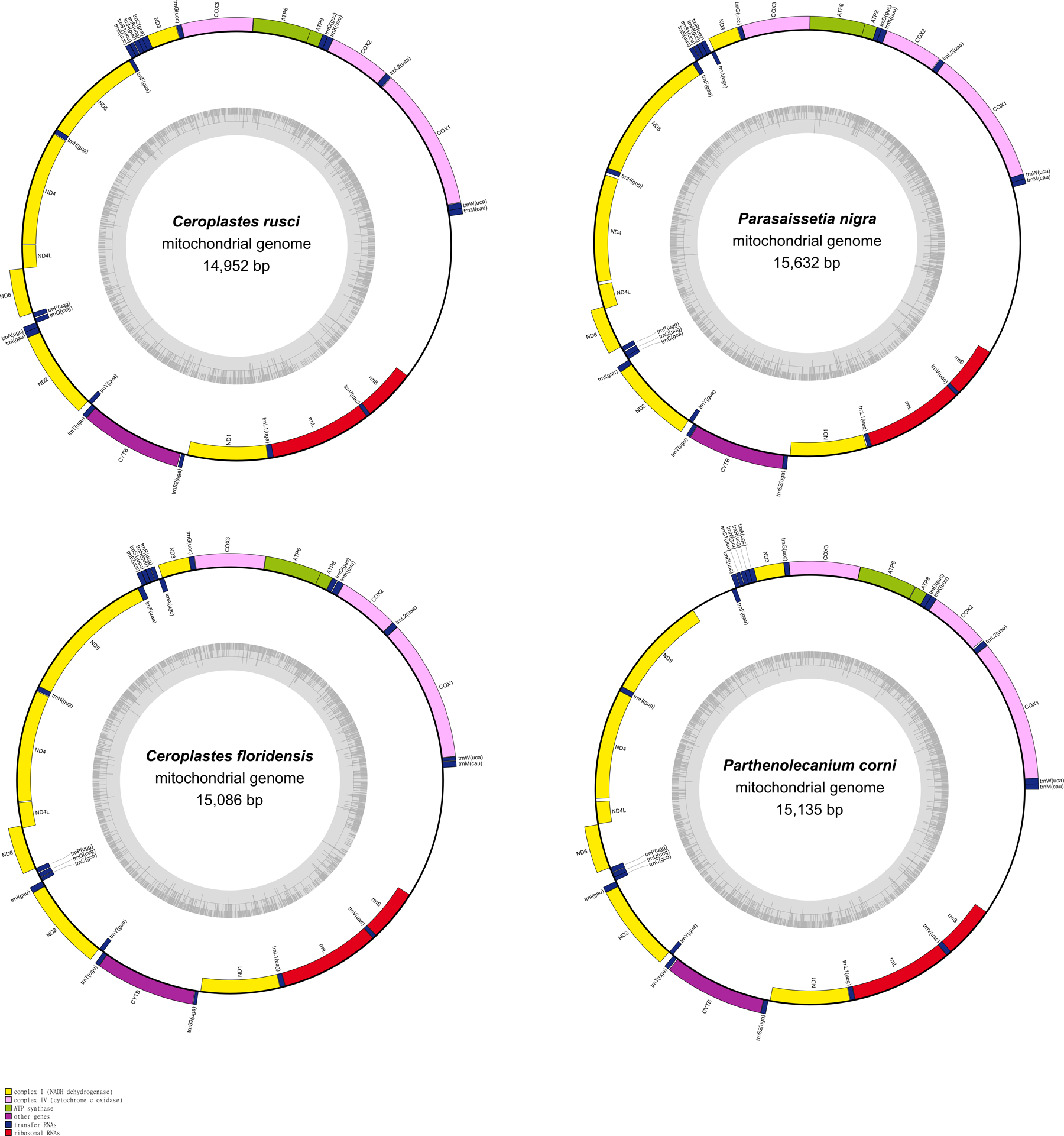

Supplement: Supplementary file 1 — Figure S1. [file ECE3-15-e71789-s007.tif]

*Ceroplastes floridensis*

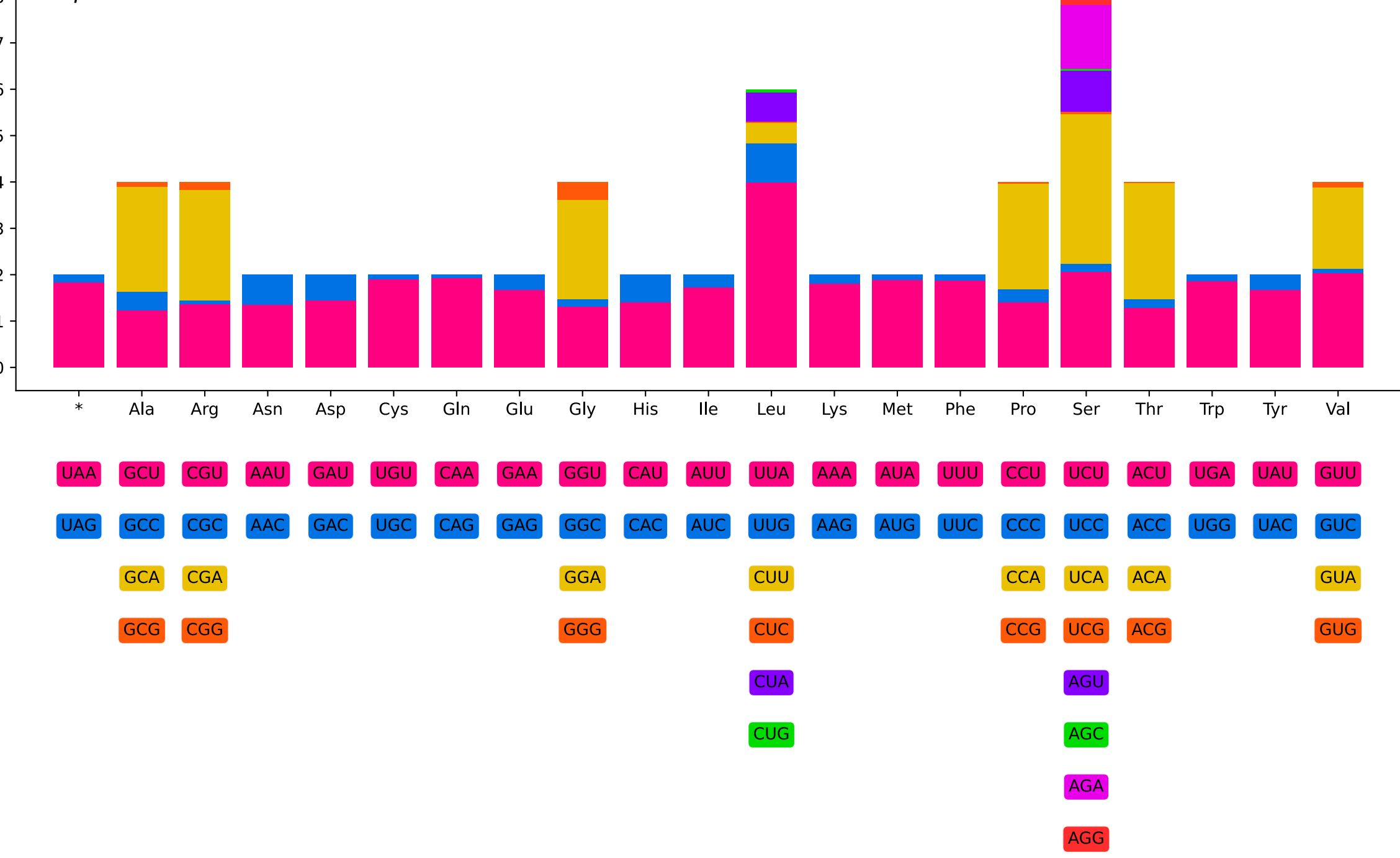

*Parasaissetia nigra*

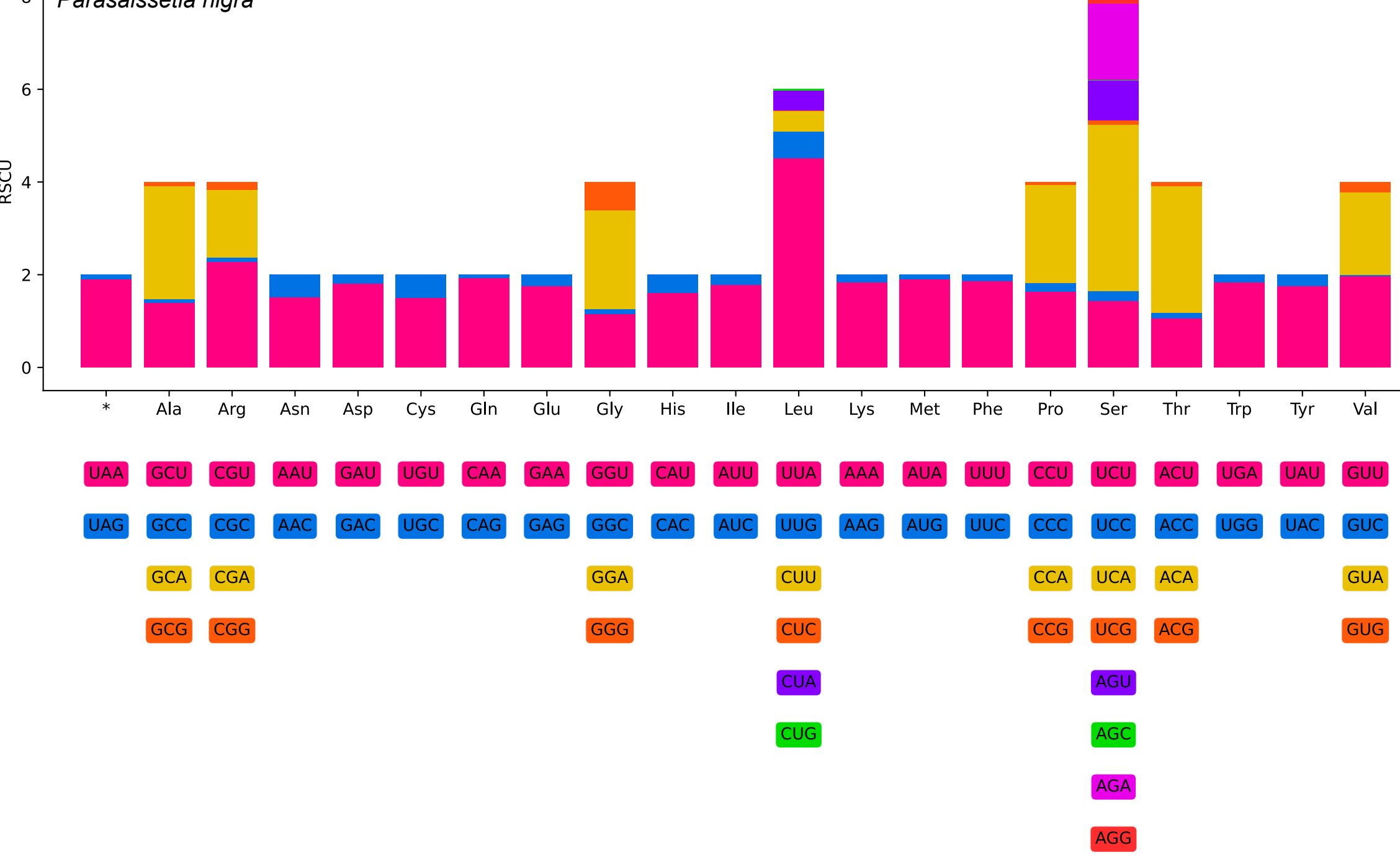

*Ceroplastes rusci*

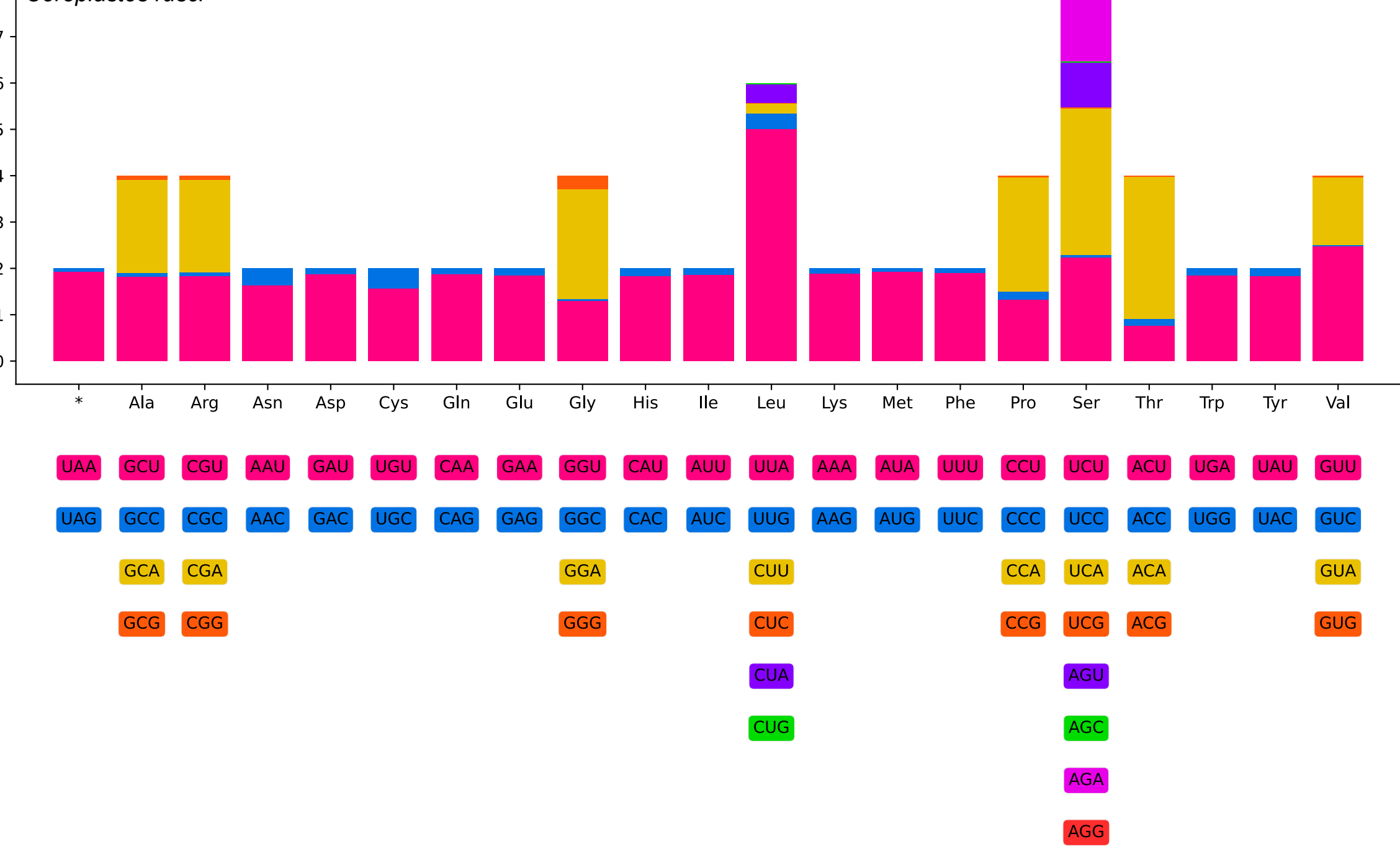

*Parthenolecanium corni*

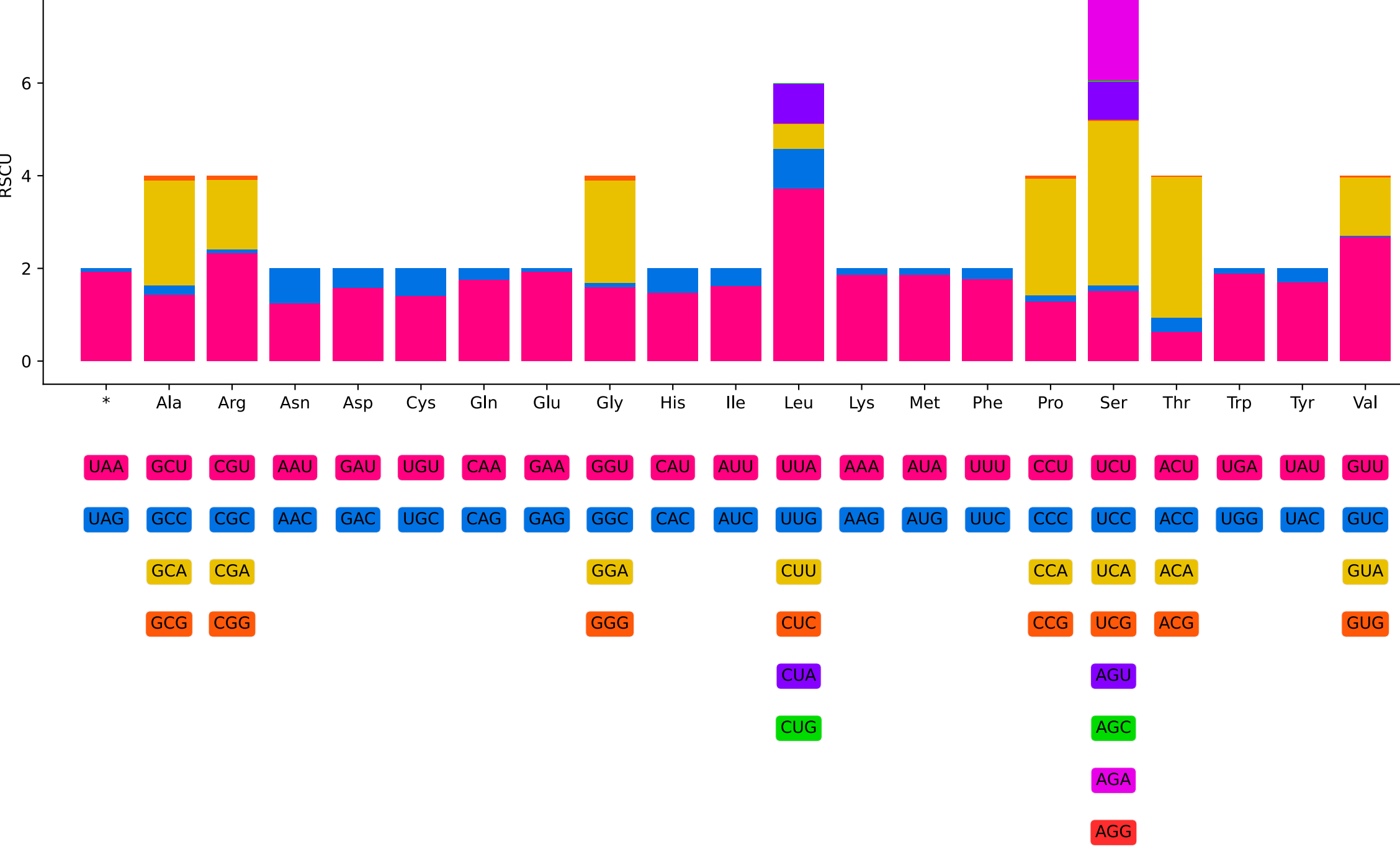

Supplement: Supplementary file 2 — Figure S2. [file ECE3-15-e71789-s011.pdf]

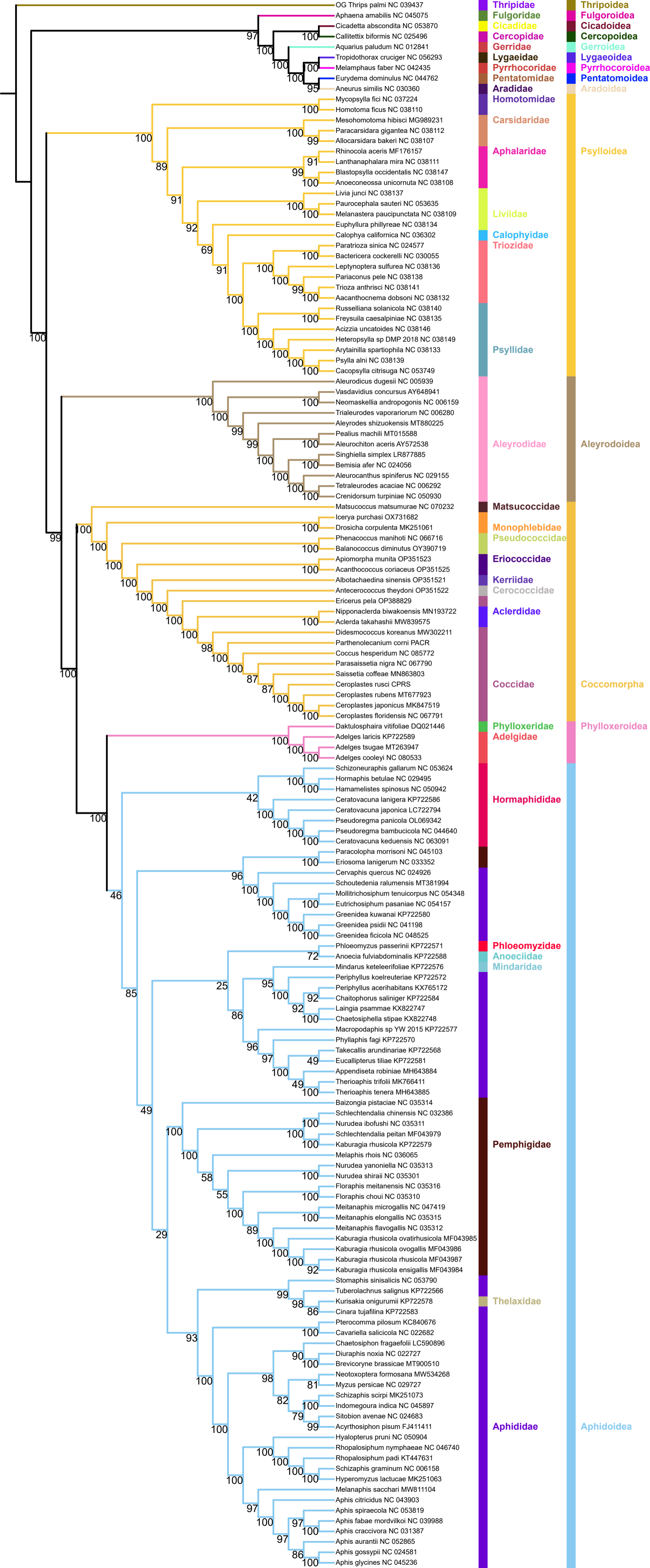

Supplement: Supplementary file 3 — Figure S3. [file ECE3-15-e71789-s005.pdf]

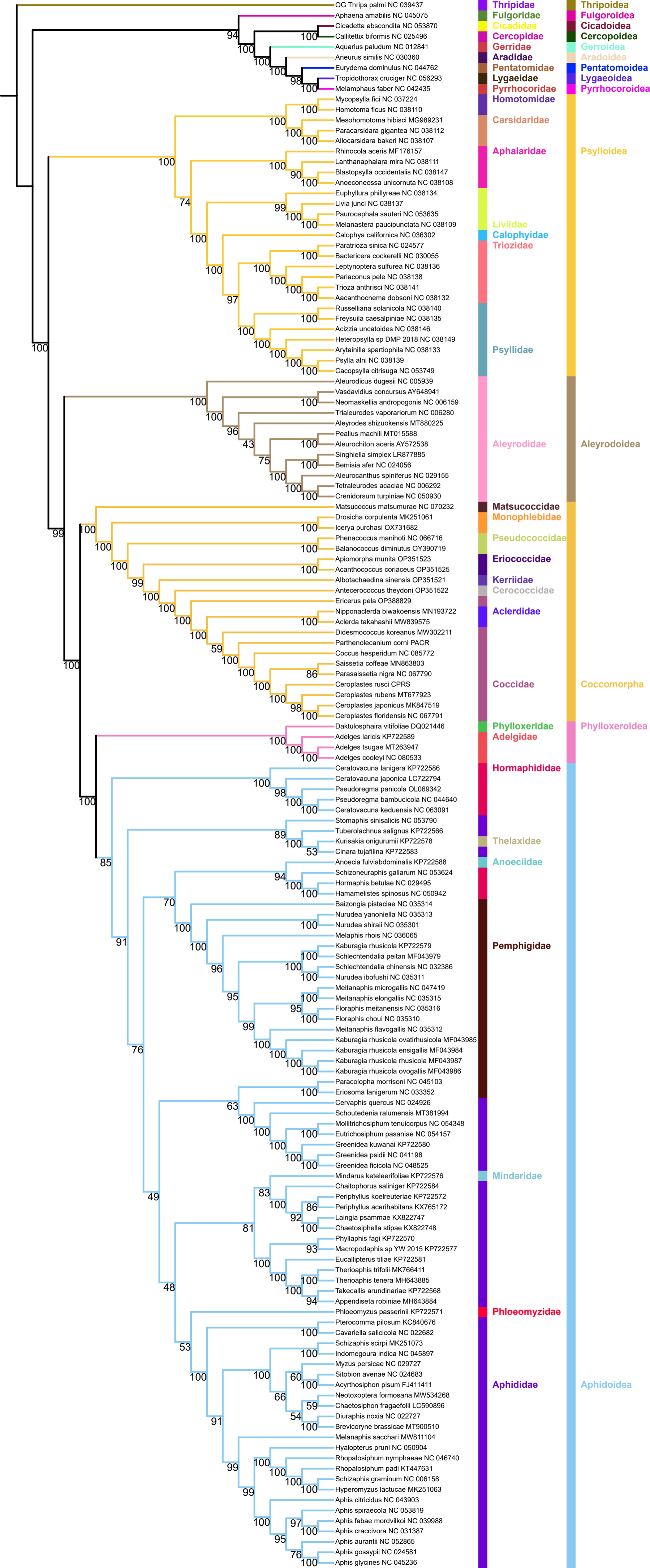

Supplement: Supplementary file 4 — Figure S4. [file ECE3-15-e71789-s004.pdf]

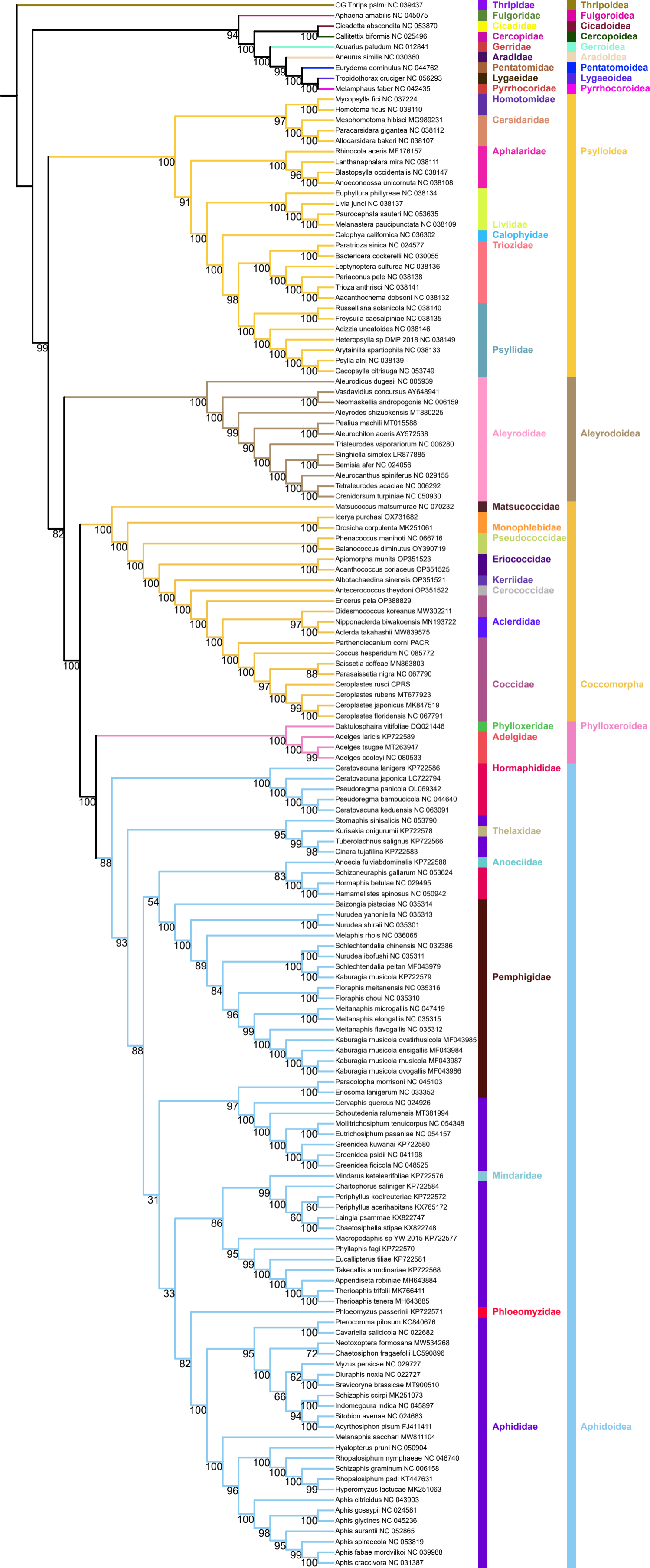

Supplement: Supplementary file 5 — Figure S5. [file ECE3-15-e71789-s010.pdf]

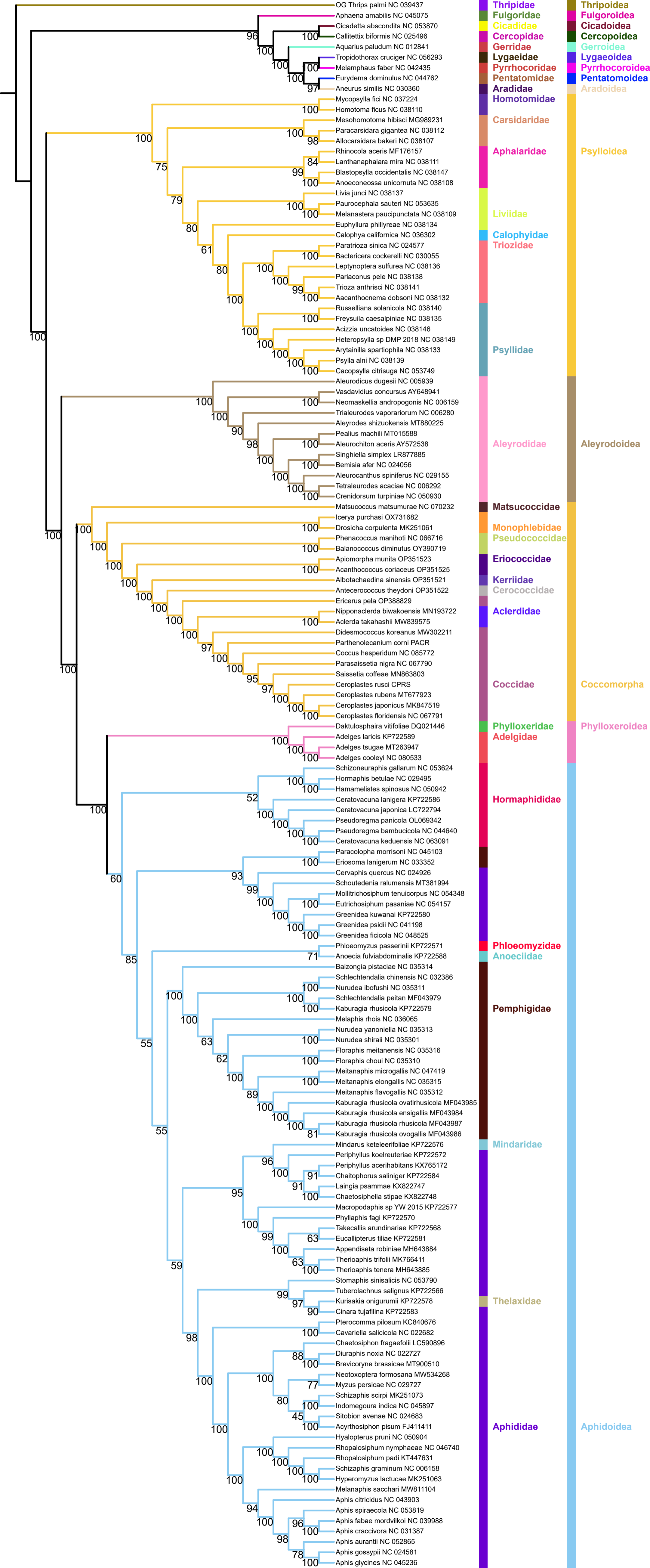

Supplement: Supplementary file 6 — Figure S6. [file ECE3-15-e71789-s008.pdf]

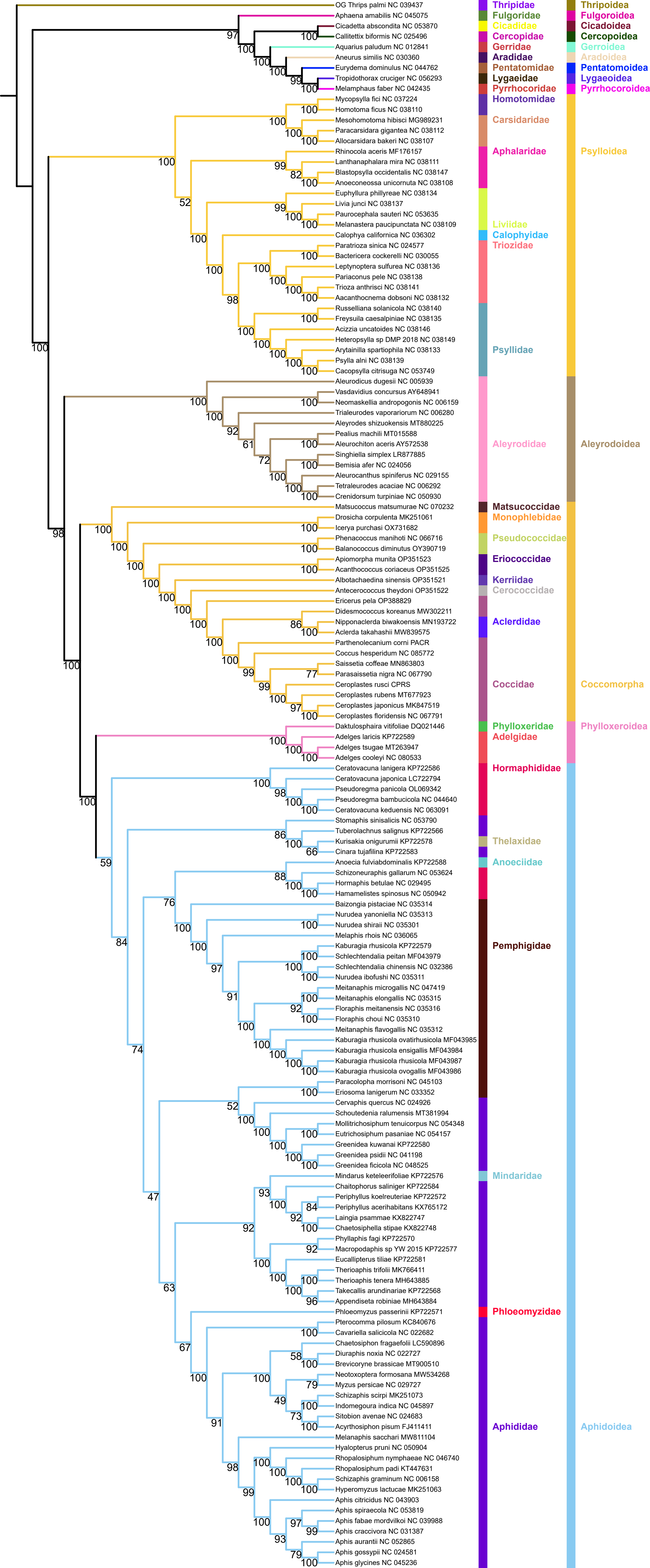

Supplement: Supplementary file 7 — Figure S7. [file ECE3-15-e71789-s001.pdf]

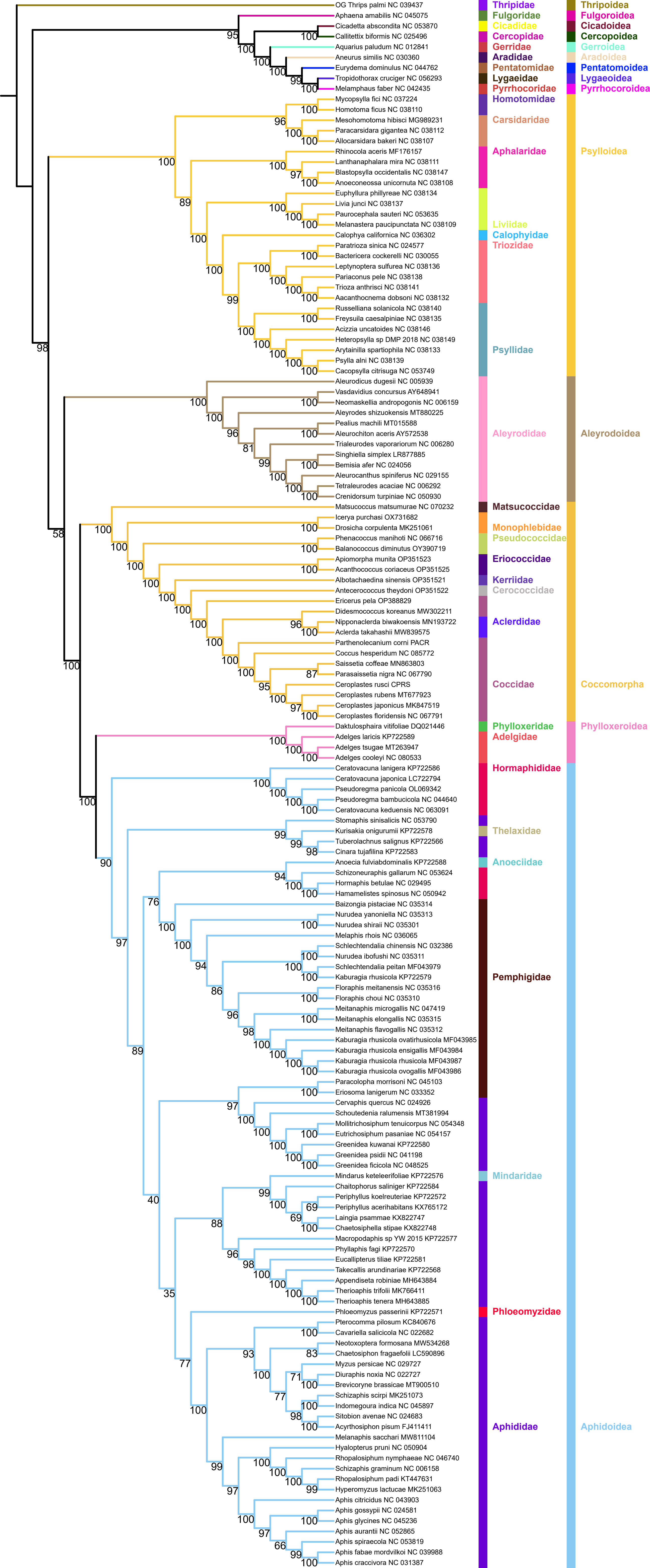

Supplement: Supplementary file 8 — Figure S8. [file ECE3-15-e71789-s006.pdf]

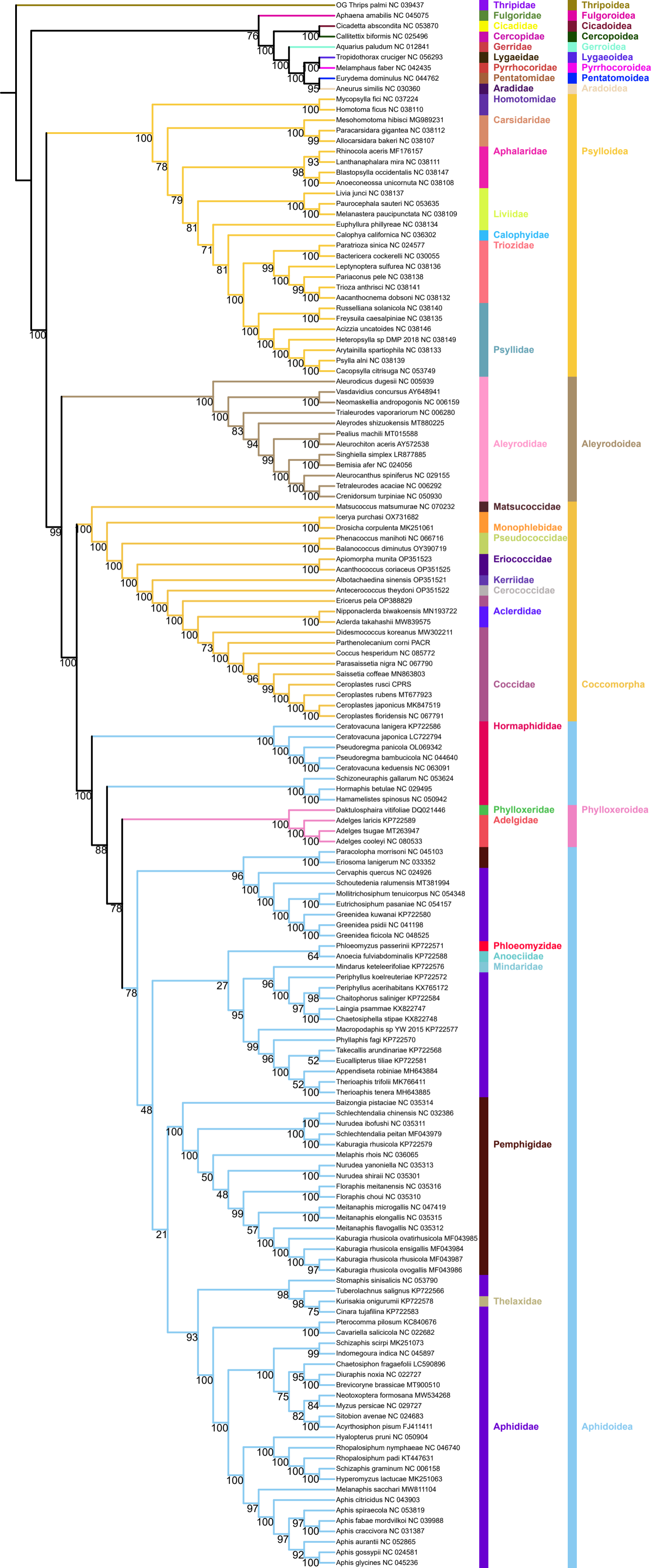

Supplement: Supplementary file 9 — Figure S9. [file ECE3-15-e71789-s013.pdf]

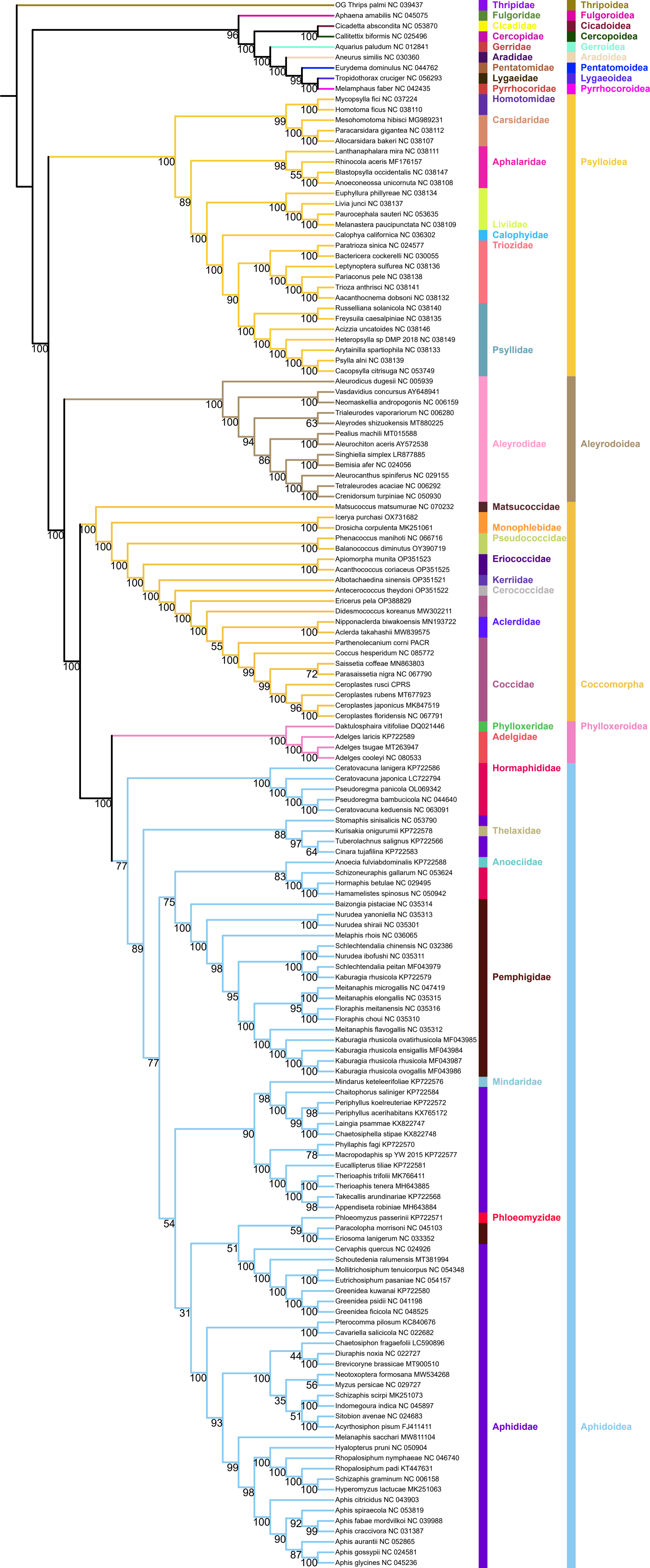

Supplement: Supplementary file 10 — Figure S10. [file ECE3-15-e71789-s009.pdf]

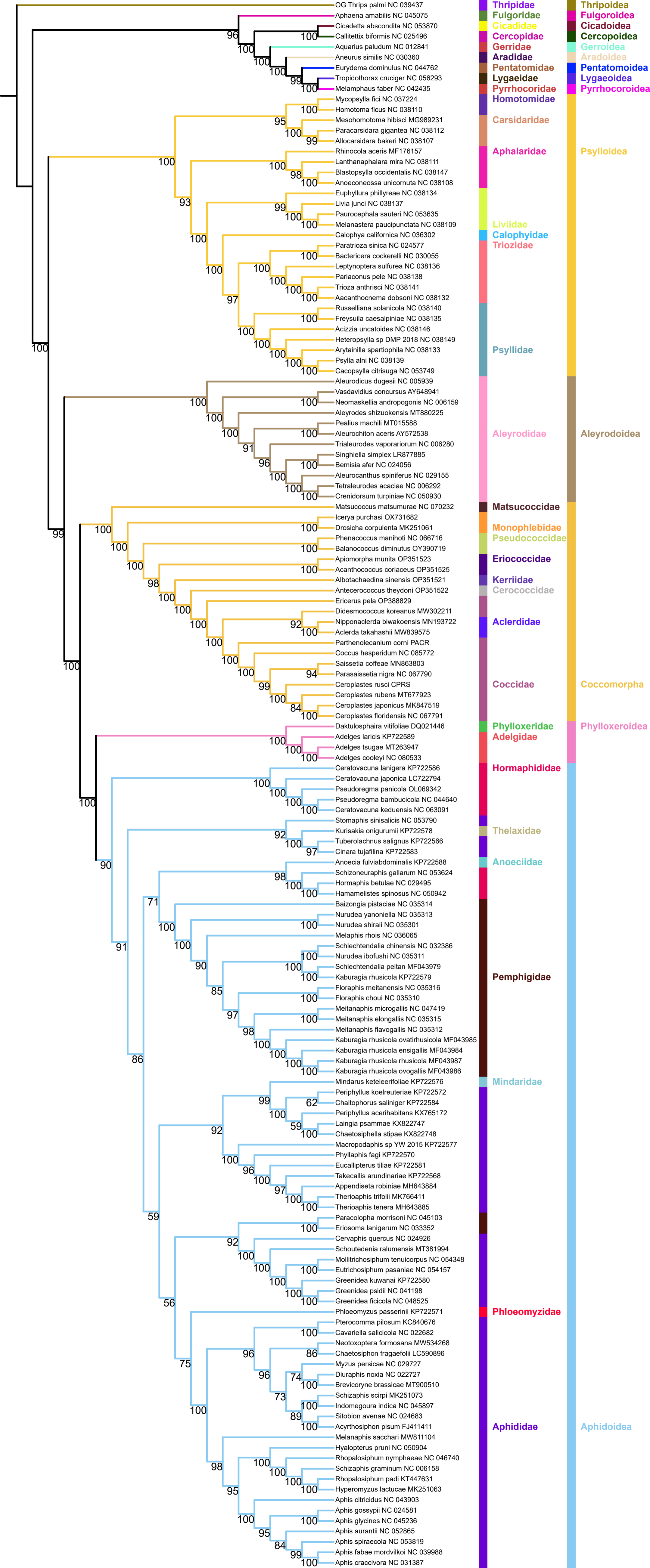

Supplement: Supplementary file 11 — Figure S11. [file ECE3-15-e71789-s002.pdf]

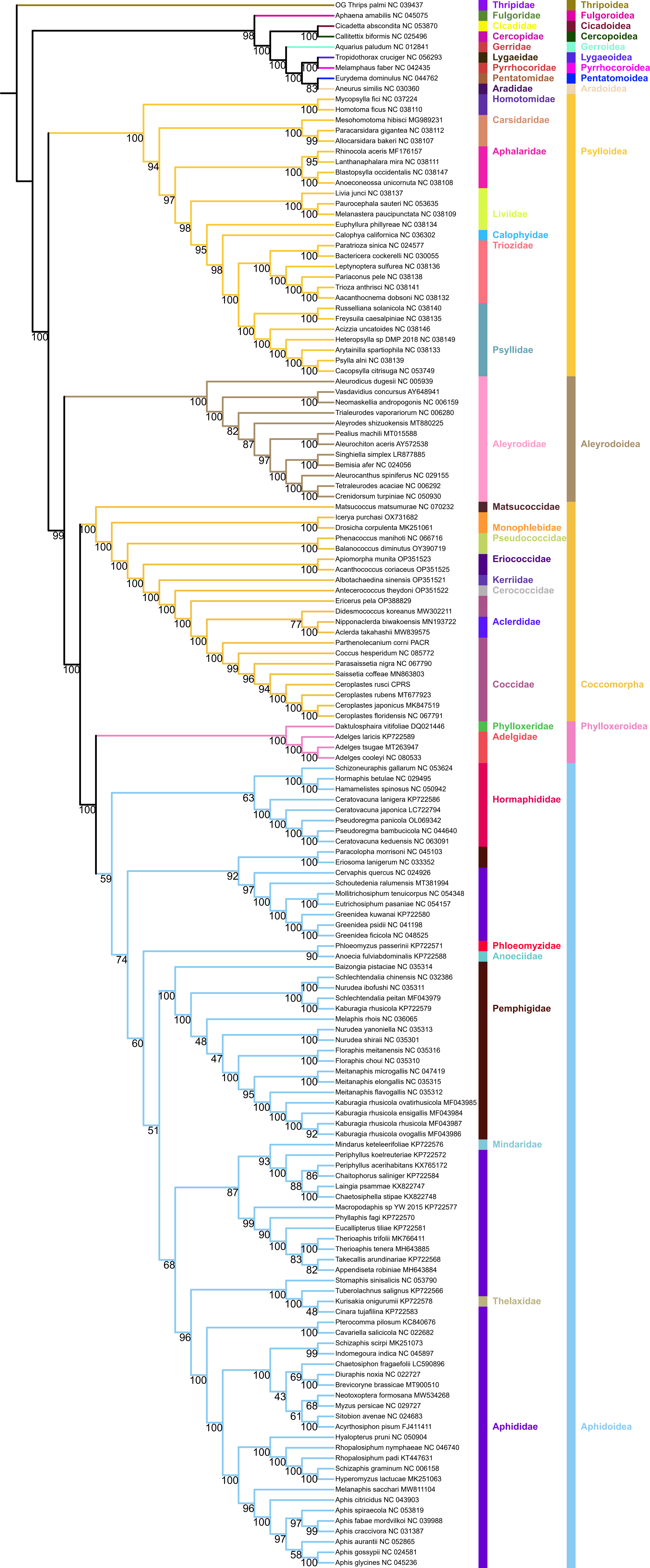

Supplement: Supplementary file 12 — Figure S12. [file ECE3-15-e71789-s012.pdf]
