# Supplementary material for: Positive Selection Drives Mitochondrial Gene Rearrangement in Sternorrhyncha (Insecta: Hemiptera)
Source: Ecol Evol. 2025 Jul 20;15(7):e71789. doi: 10.1002/ece3.71789 (PMC12277048; doi:10.1002/ece3.71789)
Supplement: Supplementary file 13 — Tables S1–S11. [file ECE3-15-e71789-s003.docx]

**Table S1** Detailed information on representatives included in the present phylogeny. The newly sequenced samples are indicated with an asterisk (*). Some species have been removed and are marked with a caret (^) sign in the selective pressures analysis.

| Superfamily | Family | Organism | Accession number |
| --- | --- | --- | --- |
| OutGroup | Aradidae | *Aneurus similis* | NC_030360 |
| OutGroup | Cercopidae | *Callitettix biformis* | NC_025496 |
| OutGroup | Cicadidae | *Cicadetta abscondita* | NC_053870 |
| OutGroup | Fulgoridae | *Aphaena amabilis* | NC_045075 |
| OutGroup | Gerridae | *Aquarius paludum* | NC_012841 |
| OutGroup | Lygaeidae | Tropidothorax cruciger | NC_056293 |
| OutGroup | Pentatomidae | *Eurydema dominulus* | NC_044762 |
| OutGroup | Pyrrhocoridae | Melamphaus faber | NC_042435 |
| OutGroup | Thripidae | *Thrips palmi* | NC_039437 |
| Aleyrodoidea | Aleyrodidae | *Aleurocanthus spiniferus* | NC_029155 |
| Aleyrodoidea | Aleyrodidae | *Aleurochiton aceris* | AY572538 |
| Aleyrodoidea | Aleyrodidae | *Aleurodicus dugesii* | NC_005939 |
| Aleyrodoidea | Aleyrodidae | *Aleyrodes shizuokensis* | MT880225 |
| Aleyrodoidea | Aleyrodidae | *Bemisia afer* | NC_024056 |
| Aleyrodoidea | Aleyrodidae | *Crenidorsum turpiniae* | NC_050930 |
| Aleyrodoidea | Aleyrodidae | *Neomaskellia andropogonis* | NC_006159 |
| Aleyrodoidea | Aleyrodidae | *Pealius machili* | MT015588 |
| Aleyrodoidea | Aleyrodidae | *Singhiella simplex* | LR877885 |
| Aleyrodoidea | Aleyrodidae | *Tetraleurodes acaciae* | NC_006292 |
| Aleyrodoidea | Aleyrodidae | *Trialeurodes vaporariorum* | NC_006280 |
| Aleyrodoidea | Aleyrodidae | *Vasdavidius concursus ^* | AY648941 |
| Aphidoidea | Adelgidae | *Adelges cooleyi* | NC_080533 |
| Aphidoidea | Adelgidae | *Adelges laricis* | KP722589 |
| Aphidoidea | Adelgidae | *Adelges tsugae* | MT263947 |
| Aphidoidea | Aphididae | *Acyrthosiphon pisum* | FJ411411 |
| Aphidoidea | Aphididae | *Anoecia fulviabdominalis* | KP722588 |
| Aphidoidea | Aphididae | *Aphis aurantii* | NC_052865 |
| Aphidoidea | Aphididae | *Aphis citricidus* | NC_043903 |
| Aphidoidea | Aphididae | *Aphis craccivora* | NC_031387 |
| Aphidoidea | Aphididae | *Aphis fabae mordvilkoi* | NC_039988 |
| Aphidoidea | Aphididae | *Aphis glycines* | NC_045236 |
| Aphidoidea | Aphididae | *Aphis gossypii* | NC_024581 |
| Aphidoidea | Aphididae | *Aphis spiraecola* | NC_053819 |
| Aphidoidea | Aphididae | *Appendiseta robiniae* | MH643884 |
| Aphidoidea | Aphididae | *Baizongia pistaciae* | NC_035314 |
| Aphidoidea | Aphididae | *Brevicoryne brassicae* | MT900510 |
| Aphidoidea | Aphididae | *Cavariella salicicola* | NC_022682 |
| Aphidoidea | Aphididae | Ceratovacuna japonica ^ | LC722794 |
| Aphidoidea | Aphididae | Ceratovacuna keduensis | NC_063091 |
| Aphidoidea | Aphididae | *Ceratovacuna lanigera ^* | KP722586 |
| Aphidoidea | Aphididae | *Cervaphis quercus* | NC_024926 |
| Aphidoidea | Aphididae | *Chaetosiphella stipae ^* | KX822748 |
| Aphidoidea | Aphididae | *Chaetosiphon fragaefolii* | LC590896 |
| Aphidoidea | Aphididae | *Chaitophorus saliniger* | KP722584 |
| Aphidoidea | Aphididae | *Cinara tujafilina ^* | KP722583 |
| Aphidoidea | Aphididae | *Diuraphis noxia* | NC_022727 |
| Aphidoidea | Aphididae | *Eriosoma lanigerum* | NC_033352 |
| Aphidoidea | Aphididae | *Eucallipterus tiliae* | KP722581 |
| Aphidoidea | Aphididae | *Eutrichosiphum pasaniae* | NC_054157 |
| Aphidoidea | Aphididae | *Floraphis choui* | NC_035310 |
| Aphidoidea | Aphididae | *Floraphis meitanensis* | NC_035316 |
| Aphidoidea | Aphididae | *Greenidea ficicola* | NC_048525 |
| Aphidoidea | Aphididae | *Greenidea kuwanai* | KP722580 |
| Aphidoidea | Aphididae | *Greenidea psidii* | NC_041198 |
| Aphidoidea | Aphididae | *Hamamelistes spinosus* | NC_050942 |
| Aphidoidea | Aphididae | *Hormaphis betulae* | NC_029495 |
| Aphidoidea | Aphididae | *Hyalopterus pruni* | NC_050904 |
| Aphidoidea | Aphididae | *Hyperomyzus lactucae* | MK251063 |
| Aphidoidea | Aphididae | *Indomegoura indica* | NC_045897 |
| Aphidoidea | Aphididae | *Kaburagia rhusicola* | KP722579 |
| Aphidoidea | Aphididae | *Kaburagia rhusicola ensigallis* | MF043984 |
| Aphidoidea | Aphididae | *Kaburagia rhusicola ovatirhusicola* | MF043985 |
| Aphidoidea | Aphididae | *Kaburagia rhusicola ovogallis* | MF043986 |
| Aphidoidea | Aphididae | *Kaburagia rhusicola rhusicola* | MF043987 |
| Aphidoidea | Aphididae | *Kurisakia onigurumii* | KP722578 |
| Aphidoidea | Aphididae | *Laingia psammae* | KX822747 |
| Aphidoidea | Aphididae | *Macropodaphis* sp. | KP722577 |
| Aphidoidea | Aphididae | *Meitanaphis elongallis* | NC_035315 |
| Aphidoidea | Aphididae | *Meitanaphis flavogallis* | NC_035312 |
| Aphidoidea | Aphididae | *Meitanaphis microgallis* | NC_047419 |
| Aphidoidea | Aphididae | *Melanaphis sacchari* | MW811104 |
| Aphidoidea | Aphididae | *Melaphis rhois* | NC_036065 |
| Aphidoidea | Aphididae | *Mindarus keteleerifoliae* | KP722576 |
| Aphidoidea | Aphididae | *Mollitrichosiphum tenuicorpus* | NC_054348 |
| Aphidoidea | Aphididae | *Myzus persicae* | NC_029727 |
| Aphidoidea | Aphididae | *Neotoxoptera formosana* | MW534268 |
| Aphidoidea | Aphididae | *Nurudea ibofushi* | NC_035311 |
| Aphidoidea | Aphididae | *Nurudea shiraii* | NC_035301 |
| Aphidoidea | Aphididae | *Nurudea yanoniella* | NC_035313 |
| Aphidoidea | Aphididae | *Paracolopha morrisoni* | NC_045103 |
| Aphidoidea | Aphididae | *Periphyllus acerihabitans ^* | KX765172 |
| Aphidoidea | Aphididae | *Periphyllus koelreuteriae* | KP722572 |
| Aphidoidea | Aphididae | *Phloeomyzus passerinii* | KP722571 |
| Aphidoidea | Aphididae | *Phyllaphis fagi ^* | KP722570 |
| Aphidoidea | Aphididae | *Pseudoregma bambucicola* | NC_044640 |
| Aphidoidea | Aphididae | Pseudoregma panicola | OL069342 |
| Aphidoidea | Aphididae | *Pterocomma pilosum ^* | KC840676 |
| Aphidoidea | Aphididae | *Rhopalosiphum nymphaeae* | NC_046740 |
| Aphidoidea | Aphididae | *Rhopalosiphum padi* | KT447631 |
| Aphidoidea | Aphididae | *Schizaphis graminum* | NC_006158 |
| Aphidoidea | Aphididae | *Schizaphis scirpi* | MK251073 |
| Aphidoidea | Aphididae | *Schizoneuraphis gallarum* | NC_053624 |
| Aphidoidea | Aphididae | *Schlechtendalia chinensis* | NC_032386 |
| Aphidoidea | Aphididae | *Schlechtendalia peitan* | MF043979 |
| Aphidoidea | Aphididae | *Schoutedenia ralumensis* | MT381994 |
| Aphidoidea | Aphididae | *Sitobion avenae* | NC_024683 |
| Aphidoidea | Aphididae | *Stomaphis sinisalicis* | NC_053790 |
| Aphidoidea | Aphididae | *Takecallis arundinariae ^* | KP722568 |
| Aphidoidea | Aphididae | *Therioaphis tenera ^* | MH643885 |
| Aphidoidea | Aphididae | *Therioaphis trifolii* | MK766411 |
| Aphidoidea | Aphididae | *Tuberolachnus salignus* | KP722566 |
| Aphidoidea | Phylloxeridae | *Daktulosphaira vitifoliae ^* | DQ021446 |
| Coccoidea | Aclerdidae | *Aclerda takahashii ^* | NC_063660 |
| Coccoidea | Aclerdidae | *Nipponaclerda biwakoensis ^* | MN193722 |
| Coccoidea | Cerococcidae | *Antecerococcus theydoni* | OP351522 |
| Coccoidea | Coccidae | *Ceroplastes floridensis ** | NC_067791 |
| Coccoidea | Coccidae | *Ceroplastes japonicus ^* | MK847519 |
| Coccoidea | Coccidae | *Ceroplastes rubens* | MT677923 |
| Coccoidea | Coccidae | *Ceroplastes rusci ** | PV269613 |
| Coccoidea | Coccidae | *Coccus hesperidum* | NC_085772 |
| Coccoidea | Coccidae | *Didesmococcus koreanus* | MW302211 |
| Coccoidea | Coccidae | *Ericerus pela ^* | ON055488 |
| Coccoidea | Coccidae | *Parasaissetia nigra ** | NC_067790 |
| Coccoidea | Coccidae | *Parthenolecanium corni ** | PV269614 |
| Coccoidea | Coccidae | *Saissetia coffeae* | MN863803 |
| Coccoidea | Eriococcidae | *Acanthococcus coriaceus* | OP351525 |
| Coccoidea | Eriococcidae | *Apiomorpha munita* | OP351523 |
| Coccoidea | Kerriidae | *Albotachaedina sinensis* | OP351521 |
| Coccoidea | Margarodidae | *Matsucoccus matsumurae* | NC_070232 |
| Coccoidea | Monophlebidae | *Drosicha corpulenta ^* | MK251061 |
| Coccoidea | Monophlebidae | *Icerya purchasi ^* | OX731682 |
| Coccoidea | Pseudococcidae | *Balanococcus diminutus ^* | OY390719 |
| Coccoidea | Pseudococcidae | *Phenacoccus manihoti* | NC_066716 |
| Psylloidea | Aphalaridae | *Anoeconeossa unicornuta* | NC_038108 |
| Psylloidea | Aphalaridae | *Blastopsylla occidentalis* | NC_038147 |
| Psylloidea | Aphalaridae | *Lanthanaphalara mira* | NC_038111 |
| Psylloidea | Aphalaridae | *Rhinocola aceris* | MF176157 |
| Psylloidea | Calophyidae | *Calophya californica* | NC_036302 |
| Psylloidea | Carsidaridae | *Allocarsidara bakeri* | NC_038107 |
| Psylloidea | Carsidaridae | *Mesohomotoma hibisci ^* | MG989231 |
| Psylloidea | Carsidaridae | *Paracarsidara gigantea* | NC_038112 |
| Psylloidea | Homotomidae | *Homotoma ficus* | NC_038110 |
| Psylloidea | Homotomidae | *Mycopsylla fici* | NC_037224 |
| Psylloidea | Liviidae | *Diclidophlebia paucipunctata* | NC_038109 |
| Psylloidea | Liviidae | *Euphyllura phillyreae* | NC_038134 |
| Psylloidea | Liviidae | *Livia junci* | NC_038137 |
| Psylloidea | Liviidae | *Paurocephala sauteri* | NC_053635 |
| Psylloidea | Psyllidae | *Acizzia uncatoides* | NC_038146 |
| Psylloidea | Psyllidae | *Arytainilla spartiophila* | NC_038133 |
| Psylloidea | Psyllidae | *Cacopsylla citrisuga* | NC_053749 |
| Psylloidea | Psyllidae | *Freysuila caesalpiniae* | NC_038135 |
| Psylloidea | Psyllidae | *Heteropsylla cubana* | NC_038149 |
| Psylloidea | Psyllidae | *Psylla alni* | NC_038139 |
| Psylloidea | Psyllidae | *Russelliana solanicola* | NC_038140 |
| Psylloidea | Triozidae | *Aacanthocnema dobsoni* | NC_038132 |
| Psylloidea | Triozidae | *Bactericera cockerelli* | NC_030055 |
| Psylloidea | Triozidae | *Leptynoptera sulfurea* | NC_038136 |
| Psylloidea | Triozidae | *Paratrioza sinica* | NC_024577 |
| Psylloidea | Triozidae | *Pariaconus pele* | NC_038138 |
| Psylloidea | Triozidae | *Trioza anthrisci* | NC_038141 |

**Table S2** Annotations and gene organization of *Ceroplastes floridensis.*

| Gene | Type | Start | End | Length | Start Codon | Stop Codon | Anticodon | Strand |
| --- | --- | --- | --- | --- | --- | --- | --- | --- |
| tRNA-Met | tRNA | 143 | 209 | 67 |  |  | cau | + |
| tRNA-Trp | tRNA | 211 | 254 | 44 |  |  | uca | + |
| *COX1* | CDS | 255 | 1781 | 1527 | ATA | TAA |  | + |
| tRNA-Leu2 | tRNA | 1781 | 1845 | 65 |  |  | uaa | + |
| *COX2* | CDS | 1846 | 2509 | 664 | ATA | T |  | + |
| tRNA-Lys | tRNA | 2510 | 2581 | 72 |  |  | uuu | + |
| tRNA-Asp | tRNA | 2588 | 2643 | 56 |  |  | guc | + |
| *ATP8* | CDS | 2644 | 2784 | 141 | ATT | TAA |  | + |
| *ATP6* | CDS | 2778 | 3389 | 612 | ATG | TAA |  | + |
| *COX3* | CDS | 3391 | 4152 | 762 | ATG | TAA |  | + |
| tRNA-Gly | tRNA | 4154 | 4210 | 57 |  |  | ucc | + |
| *ND3* | CDS | 4211 | 4546 | 336 | ATA | TAA |  | + |
| tRNA-Ala | tRNA | 4528 | 4589 | 62 |  |  | ugc | - |
| tRNA-Arg | tRNA | 4586 | 4632 | 47 |  |  | ucg | + |
| tRNA-Asn | tRNA | 4634 | 4687 | 54 |  |  | guu | + |
| tRNA-Ser1 | tRNA | 4686 | 4735 | 50 |  |  | ucu | + |
| tRNA-Glu | tRNA | 4735 | 4791 | 57 |  |  | uuc | + |
| tRNA-Phe | tRNA | 4784 | 4847 | 64 |  |  | uaa | - |
| *ND5* | CDS | 4847 | 6445 | 1599 | ATT | TAA |  | - |
| tRNA-His | tRNA | 6446 | 6501 | 56 |  |  | gug | - |
| *ND4* | CDS | 6506 | 7786 | 1281 | ATT | TAA |  | - |
| *ND4L* | CDS | 7797 | 8087 | 291 | ATT | TAA |  | - |
| *ND6* | CDS | 8054 | 8563 | 510 | ATA | TAA |  | + |
| tRNA-Pro | tRNA | 8562 | 8619 | 58 |  |  | ugg | - |
| tRNA-Gln | tRNA | 8626 | 8680 | 55 |  |  | uug | - |
| tRNA-Cys | tRNA | 8682 | 8726 | 45 |  |  | gca | - |
| tRNA-Ile | tRNA | 8724 | 8788 | 65 |  |  | gau | + |
| *ND2* | CDS | 8789 | 9727 | 939 | ATT | TAA |  | + |
| tRNA-Tyr | tRNA | 9729 | 9784 | 56 |  |  | gua | - |
| tRNA-Thr | tRNA | 9788 | 9838 | 51 |  |  | ugu | + |
| *CYTB* | CDS | 9839 | 10918 | 1080 | ATA | TAA |  | + |
| tRNA-Ser2 | tRNA | 10921 | 10959 | 39 |  |  | uga | + |
| *ND1* | CDS | 10981 | 11886 | 906 | ATA | TAG |  | - |
| tRNA-Leu1 | tRNA | 11888 | 11944 | 57 |  |  | uag | - |
| *rrnL* | rRNA | 11945 | 13084 | 1140 |  |  |  | - |
| tRNA-Val | tRNA | 13085 | 13129 | 45 |  |  | uac | - |
| *rrnS* | rRNA | 13130 | 13728 | 599 |  |  |  | - |

**Table S3** Annotations and gene organization of *Ceroplastes rusci.*

| Gene | Type | Start | End | Length | Start Codon | Stop Codon | Anticodon | Strand |
| --- | --- | --- | --- | --- | --- | --- | --- | --- |
| tRNA-Met | tRNA | 332 | 403 | 72 |  |  | cau | + |
| tRNA-Trp | tRNA | 394 | 456 | 63 |  |  | uca | + |
| *COX1* | CDS | 447 | 1971 | 1525 | ATT | T |  | + |
| tRNA-Leu2 | tRNA | 1975 | 2038 | 64 |  |  | uaa | + |
| *COX2* | CDS | 2039 | 2699 | 661 | ATA | T |  | + |
| tRNA-Lys | tRNA | 2700 | 2764 | 65 |  |  | uuu | + |
| tRNA-Asp | tRNA | 2761 | 2817 | 57 |  |  | guc | + |
| *ATP8* | CDS | 2814 | 2954 | 141 | ATT | TAA |  | + |
| *ATP6* | CDS | 2948 | 3562 | 615 | ATG | TAA |  | + |
| *COX3* | CDS | 3563 | 4324 | 762 | ATG | TAA |  | + |
| tRNA-Gly | tRNA | 4325 | 4375 | 51 |  |  | tcc | + |
| *ND3* | CDS | 4378 | 4716 | 339 | ATA | TAA |  | + |
| tRNA-Cys | tRNA | 4693 | 4758 | 66 |  |  | gca | + |
| tRNA-Arg | tRNA | 4753 | 4802 | 50 |  |  | ucg | + |
| tRNA-Asn | tRNA | 4795 | 4850 | 56 |  |  | guu | + |
| tRNA-Ser1 | tRNA | 4854 | 4899 | 46 |  |  | ucu | + |
| tRNA-Glu | tRNA | 4898 | 4952 | 55 |  |  | uuc | + |
| tRNA-Phe | tRNA | 4944 | 4988 | 45 |  |  | gaa | - |
| *ND5* | CDS | 4989 | 6107 | 1119 | ATA | TAA |  | - |
| tRNA-His | tRNA | 6105 | 6161 | 57 |  |  | gug | - |
| *ND4* | CDS | 6166 | 7455 | 1290 | ATT | TAA |  | - |
| *ND4L* | CDS | 7460 | 7726 | 267 | ATT | TAA |  | - |
| *ND6* | CDS | 7725 | 8231 | 507 | ATG | TAA |  | + |
| tRNA-Pro | tRNA | 8228 | 8284 | 57 |  |  | ugg | - |
| tRNA-Gln | tRNA | 8293 | 8349 | 57 |  |  | uug | - |
| tRNA-Ala | tRNA | 8340 | 8398 | 59 |  |  | ugc | + |
| tRNA-Ile | tRNA | 8395 | 8458 | 64 |  |  | gau | + |
| *ND2* | CDS | 8459 | 9397 | 939 | ATA | TAA |  | + |
| tRNA-Tyr | tRNA | 9395 | 9448 | 54 |  |  | gua | - |
| tRNA-Thr | tRNA | 9451 | 9505 | 55 |  |  | ugu | + |
| *CYTB* | CDS | 9506 | 10585 | 1080 | ATA | TAA |  | + |
| tRNA-Ser2 | tRNA | 10593 | 10637 | 45 |  |  | uga | + |
| *ND1* | CDS | 10661 | 11563 | 903 | ATT | TAA |  | - |
| tRNA-Leu1 | tRNA | 11565 | 11627 | 63 |  |  | uga | - |
| *rrnL* | rRNA | 11628 | 12768 | 1141 |  |  |  | - |
| tRNA-Val | tRNA | 12769 | 12813 | 45 |  |  | uac | - |
| *rrnS* | rRNA | 12814 | 13408 | 595 |  |  |  | - |

**Table S4** Annotations and gene organization of *Parasaissetia nigra.*

| Gene | Type | Start | End | Length | Start Codon | Stop Codon | Anticodon | Strand |
| --- | --- | --- | --- | --- | --- | --- | --- | --- |
| tRNA-Met | tRNA | 659 | 721 | 63 |  |  | cau | + |
| tRNA-Trp | tRNA | 713 | 774 | 62 |  |  | uca | + |
| *COX1* | CDS | 765 | 2289 | 1525 | ATT | T |  | + |
| tRNA-Leu2 | tRNA | 2290 | 2352 | 63 |  |  | uaa | + |
| *COX2* | CDS | 2356 | 3013 | 658 | ATA | T |  | + |
| tRNA-Lys | tRNA | 3014 | 3081 | 68 |  |  | uuu | + |
| tRNA-Asp | tRNA | 3077 | 3132 | 56 |  |  | guc | + |
| *ATP8* | CDS | 3129 | 3269 | 141 | ATT | TAA |  | + |
| *ATP6* | CDS | 3263 | 3877 | 615 | ATG | TAA |  | + |
| *COX3* | CDS | 3877 | 4638 | 762 | ATG | TAA |  | + |
| tRNA-Gly | tRNA | 4638 | 4687 | 50 |  |  | ucc | + |
| *ND3* | CDS | 4690 | 5028 | 339 | ATA | TAA |  | + |
| tRNA-Ala | tRNA | 5023 | 5073 | 51 |  |  | ugc | - |
| tRNA-Arg | tRNA | 5070 | 5122 | 53 |  |  | ucg | + |
| tRNA-Asn | tRNA | 5119 | 5171 | 53 |  |  | guu | + |
| tRNA-Ser1 | tRNA | 5171 | 5219 | 49 |  |  | ucu | + |
| tRNA-Glu | tRNA | 5221 | 5270 | 50 |  |  | uuc | + |
| tRNA-Phe | tRNA | 5263 | 5321 | 59 |  |  | gaa | - |
| *ND5* | CDS | 5322 | 6921 | 1600 | ATT | T |  | - |
| tRNA-His | tRNA | 6922 | 6977 | 56 |  |  | gug | - |
| *ND4* | CDS | 7004 | 8278 | 1275 | ATG | TAA |  | - |
| *ND4L* | CDS | 8309 | 8599 | 291 | ATT | TAA |  | - |
| *ND6* | CDS | 8566 | 9081 | 516 | ATA | TAA |  | + |
| tRNA-Pro | tRNA | 9083 | 9140 | 58 |  |  | ugg | - |
| tRNA-Gln | tRNA | 9152 | 9221 | 70 |  |  | uug | - |
| tRNA-Cys | tRNA | 9209 | 9251 | 43 |  |  | gca | - |
| tRNA-Ile | tRNA | 9254 | 9318 | 65 |  |  | gau | + |
| *ND2* | CDS | 9319 | 10257 | 939 | ATA | TAA |  | + |
| tRNA-Tyr | tRNA | 10255 | 10310 | 56 |  |  | gua | - |
| tRNA-Thr | tRNA | 10324 | 10373 | 50 |  |  | ugu | + |
| *CYTB* | CDS | 10375 | 11451 | 1077 | ATT | TAA |  | + |
| tRNA-Ser2 | tRNA | 11451 | 11499 | 49 |  |  | uga | + |
| *ND1* | CDS | 11522 | 12430 | 909 | ATT | TAA |  | - |
| tRNA-Leu1 | tRNA | 12440 | 12489 | 50 |  |  | uag | - |
| *rrnL* | rRNA | 12491 | 13654 | 1164 |  |  |  | - |
| tRNA-Val | tRNA | 13655 | 13700 | 46 |  |  | uac | - |
| *rrnS* | rRNA | 13701 | 14295 | 595 |  |  |  | - |

**Table S5** Annotations and gene organization of *Parthenolecanium corni.*

| Gene | Type | Start | End | Length | Start Codon | Stop Codon | Anticodon | Strand |
| --- | --- | --- | --- | --- | --- | --- | --- | --- |
| tRNA-Met | tRNA | 1 | 69 | 69 |  |  | cau | + |
| tRNA-Trp | tRNA | 61 | 126 | 66 |  |  | uca | + |
| *COX1* | CDS | 117 | 1646 | 1530 | ATT | TAA |  | + |
| tRNA-Leu2 | tRNA | 1647 | 1709 | 63 |  |  | uaa | + |
| *COX2* | CDS | 1722 | 2373 | 652 | ATA | T |  | + |
| tRNA-Lys | tRNA | 2374 | 2436 | 63 |  |  | uuu | + |
| tRNA-Asp | tRNA | 2432 | 2490 | 59 |  |  | guc | + |
| *ATP8* | CDS | 2487 | 2627 | 141 | ATT | TAA |  | + |
| *ATP6* | CDS | 2629 | 3246 | 618 | ATA | TAA |  | + |
| *COX3* | CDS | 3246 | 4007 | 762 | ATG | TAA |  | + |
| tRNA-Gly | tRNA | 4007 | 4063 | 57 |  |  | ucc | + |
| *ND3* | CDS | 4061 | 4399 | 339 | ATA | TAA |  | + |
| tRNA-Ala | tRNA | 4381 | 4440 | 60 |  |  | ugc | + |
| tRNA-Arg | tRNA | 4436 | 4484 | 49 |  |  | ucg | + |
| tRNA-Asn | tRNA | 4476 | 4529 | 54 |  |  | guu | + |
| tRNA-Ser1 | tRNA | 4533 | 4578 | 46 |  |  | ucu | + |
| tRNA-Glu | tRNA | 4581 | 4635 | 55 |  |  | uuc | + |
| tRNA-Phe | tRNA | 4630 | 4685 | 56 |  |  | gaa | - |
| *ND5* | CDS | 5175 | 6359 | 1185 | ATT | TAA |  | - |
| tRNA-His | tRNA | 6360 | 6415 | 56 |  |  | gug | - |
| *ND4* | CDS | 6396 | 7667 | 1272 | ATG | TAA |  | - |
| *ND4L* | CDS | 7700 | 7954 | 255 | ATT | TAA |  | - |
| *ND6* | CDS | 7957 | 8463 | 507 | ATT | TAA |  | + |
| tRNA-Pro | tRNA | 8461 | 8521 | 61 |  |  | ugg | - |
| tRNA-Gln | tRNA | 8524 | 8580 | 57 |  |  | uug | - |
| tRNA-Cys | tRNA | 8577 | 8622 | 46 |  |  | gca | - |
| tRNA-Ile | tRNA | 8627 | 8690 | 64 |  |  | gau | + |
| *ND2* | CDS | 8691 | 9629 | 939 | ATA | TAA |  | + |
| tRNA-Tyr | tRNA | 9627 | 9680 | 54 |  |  | gua | - |
| tRNA-Thr | tRNA | 9689 | 9741 | 53 |  |  | ugu | + |
| *CYTB* | CDS | 9750 | 10826 | 1077 | ATG | TAA |  | + |
| tRNA-Ser2 | tRNA | 10827 | 10881 | 55 |  |  | uga | + |
| *ND1* | CDS | 10898 | 11803 | 906 | ATT | TAA |  | - |
| tRNA-Leu1 | tRNA | 11806 | 11861 | 56 |  |  | uag | - |
| *rrnL* | rRNA | 11862 | 13016 | 1155 |  |  |  | - |
| tRNA-Val | tRNA | 13017 | 13079 | 63 |  |  | uac | - |
| *rrnS* | rRNA | 13080 | 13678 | 599 |  |  |  | - |

**Table S6** Base composition analysis of four scale insects

| Gene | *Ceroplastes rusci* | *Ceroplastes floridensis* | *Parthenolecanium corni* | *Parasaissetia nigra* |
| --- | --- | --- | --- | --- |
| *ATP6* | 88.62 | 84.97 | 86.57 | 88.29 |
| *ATP8* | 89.36 | 86.52 | 89.36 | 90.07 |
| *COX1* | 81.05 | 77.80 | 78.04 | 78.36 |
| *COX2* | 80.19 | 80.42 | 80.64 | 80.40 |
| *COX3* | 86.48 | 85.04 | 83.73 | 86.48 |
| *CYTB* | 84.81 | 82.31 | 81.24 | 83.47 |
| *ND1* | 84.83 | 85.21 | 84.44 | 84.27 |
| *ND2* | 91.27 | 88.29 | 88.71 | 89.35 |
| *ND3* | 90.27 | 87.20 | 87.02 | 89.97 |
| *ND4* | 89.77 | 86.96 | 85.93 | 86.82 |
| *ND4L* | 92.13 | **90.03** | **90.98** | 89.69 |
| *ND5* | 86.33 | 85.37 | 82.87 | 87.88 |
| *ND6* | **92.90** | 89.61 | 89.55 | **92.25** |

**Table S7** The RSCU and codon statistics of *Ceroplastes floridensis*. The asterisk represents termination codon.

| AA | Codon | Count | RSCU | AA | Codon | Count | RSCU |
| --- | --- | --- | --- | --- | --- | --- | --- |
| * | TAA | 22 | 1.83 | Leu | TTA | 500 | 4.00 |
| * | TAG | 2 | 0.17 | Leu | TTG | 104 | 0.83 |
| Ala | GCA | 22 | 2.29 | Lys | AAA | 396 | 1.81 |
| Ala | GCC | 4 | 0.42 | Lys | AAG | 42 | 0.19 |
| Ala | GCG | 0 | 0.05 | Met | ATA | 1116 | 1.89 |
| Ala | GCT | 12 | 1.25 | Met | ATG | 66 | 0.11 |
| Arg | CGA | 28 | 2.41 | Phe | TTC | 58 | 0.12 |
| Arg | CGC | 0 | 0.04 | Phe | TTT | 902 | 1.88 |
| Arg | CGG | 2 | 0.17 | Pro | CCA | 78 | 2.29 |
| Arg | CGT | 16 | 1.38 | Pro | CCC | 10 | 0.29 |
| Asn | AAC | 200 | 0.66 | Pro | CCG | 0 | 0.02 |
| Asn | AAT | 410 | 1.34 | Pro | CCT | 48 | 1.41 |
| Asp | GAC | 28 | 0.56 | Ser | AGA | 94 | 1.39 |
| Asp | GAT | 72 | 1.44 | Ser | AGC | 2 | 0.03 |
| Cys | TGC | 2 | 0.10 | Ser | AGG | 12 | 0.18 |
| Cys | TGT | 40 | 1.91 | Ser | AGT | 60 | 0.89 |
| Gln | CAA | 58 | 1.93 | Ser | TCA | 218 | 3.22 |
| Gln | CAG | 2 | 0.07 | Ser | TCC | 12 | 0.18 |
| Glu | GAA | 100 | 1.67 | Ser | TCG | 4 | 0.06 |
| Glu | GAG | 20 | 0.33 | Ser | TCT | 140 | 2.07 |
| Gly | GGA | 78 | 2.14 | Thr | ACA | 168 | 2.52 |
| Gly | GGC | 6 | 0.16 | Thr | ACC | 12 | 0.18 |
| Gly | GGG | 14 | 0.38 | Thr | ACG | 0 | 0.01 |
| Gly | GGT | 48 | 1.32 | Thr | ACT | 86 | 1.29 |
| His | CAC | 22 | 0.60 | Trp | TGA | 110 | 1.86 |
| His | CAT | 52 | 1.41 | Trp | TGG | 8 | 0.14 |
| Ile | ATC | 114 | 0.28 | Tyr | TAC | 54 | 0.33 |
| Ile | ATT | 714 | 1.73 | Tyr | TAT | 278 | 1.68 |
| Leu | CTA | 80 | 0.64 | Val | GTA | 126 | 1.76 |
| Leu | CTC | 2 | 0.02 | Val | GTC | 6 | 0.08 |
| Leu | CTG | 8 | 0.06 | Val | GTG | 8 | 0.11 |
| Leu | CTT | 56 | 0.45 | Val | GTT | 146 | 2.04 |

**Table S8** The RSCU and codon statistics of *Ceroplastes rusci*. The asterisk represents termination codon.

| AA | Codon | Count | RSCU | AA | Codon | Count | RSCU |
| --- | --- | --- | --- | --- | --- | --- | --- |
| * | TAA | 12 | 1.92 | Leu | TTA | 212 | 3.70 |
| * | TAG | 0 | 0.08 | Leu | TTG | 50 | 0.87 |
| Ala | GCA | 11 | 2.26 | Lys | AAA | 200 | 1.86 |
| Ala | GCC | 1 | 0.21 | Lys | AAG | 15 | 0.14 |
| Ala | GCG | 0 | 0.10 | Met | ATA | 580 | 1.87 |
| Ala | GCT | 7 | 1.44 | Met | ATG | 42 | 0.14 |
| Arg | CGA | 9 | 1.50 | Phe | TTC | 52 | 0.24 |
| Arg | CGC | 0 | 0.08 | Phe | TTT | 382 | 1.76 |
| Arg | CGG | 0 | 0.08 | Pro | CCA | 41 | 2.52 |
| Arg | CGT | 14 | 2.33 | Pro | CCC | 2 | 0.12 |
| Asn | AAC | 111 | 0.75 | Pro | CCG | 1 | 0.06 |
| Asn | AAT | 185 | 1.25 | Pro | CCT | 21 | 1.29 |
| Asp | GAC | 10 | 0.43 | Ser | AGA | 61 | 1.81 |
| Asp | GAT | 37 | 1.57 | Ser | AGC | 0 | 0.02 |
| Cys | TGC | 6 | 0.60 | Ser | AGG | 5 | 0.15 |
| Cys | TGT | 14 | 1.40 | Ser | AGT | 28 | 0.83 |
| Gln | CAA | 29 | 1.76 | Ser | TCA | 120 | 3.56 |
| Gln | CAG | 4 | 0.24 | Ser | TCC | 4 | 0.12 |
| Glu | GAA | 53 | 1.93 | Ser | TCG | 0 | 0.02 |
| Glu | GAG | 2 | 0.07 | Ser | TCT | 51 | 1.51 |
| Gly | GGA | 43 | 2.21 | Thr | ACA | 91 | 3.05 |
| Gly | GGC | 2 | 0.10 | Thr | ACC | 9 | 0.30 |
| Gly | GGG | 2 | 0.10 | Thr | ACG | 0 | 0.02 |
| Gly | GGT | 31 | 1.59 | Thr | ACT | 19 | 0.64 |
| His | CAC | 9 | 0.53 | Trp | TGA | 50 | 1.89 |
| His | CAT | 25 | 1.47 | Trp | TGG | 3 | 0.11 |
| Ile | ATC | 69 | 0.38 | Tyr | TAC | 24 | 0.30 |
| Ile | ATT | 295 | 1.62 | Tyr | TAT | 137 | 1.70 |
| Leu | CTA | 50 | 0.87 | Val | GTA | 41 | 1.24 |
| Leu | CTC | 0 | 0.01 | Val | GTC | 1 | 0.03 |
| Leu | CTG | 0 | 0.01 | Val | GTG | 1 | 0.03 |
| Leu | CTT | 31 | 0.54 | Val | GTT | 89 | 2.70 |

**Table S9** The RSCU and codon statistics of *Parasaissetia nigra*. The asterisk represents termination codon.

| AA | Codon | Count | RSCU | AA | Codon | Count | RSCU |
| --- | --- | --- | --- | --- | --- | --- | --- |
| * | TAA | 20 | 1.95 | Leu | TTA | 634 | 4.52 |
| * | TAG | 0 | 0.05 | Leu | TTG | 80 | 0.57 |
| Ala | GCA | 28 | 2.49 | Lys | AAA | 410 | 1.84 |
| Ala | GCC | 0 | 0.04 | Lys | AAG | 36 | 0.16 |
| Ala | GCG | 0 | 0.04 | Met | ATA | 1142 | 1.90 |
| Ala | GCT | 16 | 1.42 | Met | ATG | 60 | 0.10 |
| Arg | CGA | 18 | 1.49 | Phe | TTC | 68 | 0.15 |
| Arg | CGC | 0 | 0.04 | Phe | TTT | 862 | 1.85 |
| Arg | CGG | 2 | 0.17 | Pro | CCA | 70 | 2.12 |
| Arg | CGT | 28 | 2.31 | Pro | CCC | 6 | 0.18 |
| Asn | AAC | 146 | 0.49 | Pro | CCG | 2 | 0.06 |
| Asn | AAT | 450 | 1.51 | Pro | CCT | 54 | 1.64 |
| Asp | GAC | 10 | 0.19 | Ser | AGA | 110 | 1.65 |
| Asp | GAT | 96 | 1.81 | Ser | AGC | 0 | 0.01 |
| Cys | TGC | 8 | 0.50 | Ser | AGG | 10 | 0.15 |
| Cys | TGT | 24 | 1.50 | Ser | AGT | 58 | 0.87 |
| Gln | CAA | 56 | 1.93 | Ser | TCA | 240 | 3.59 |
| Gln | CAG | 2 | 0.07 | Ser | TCC | 14 | 0.21 |
| Glu | GAA | 112 | 1.75 | Ser | TCG | 6 | 0.09 |
| Glu | GAG | 16 | 0.25 | Ser | TCT | 96 | 1.44 |
| Gly | GGA | 78 | 2.14 | Thr | ACA | 180 | 2.73 |
| Gly | GGC | 4 | 0.11 | Thr | ACC | 8 | 0.12 |
| Gly | GGG | 22 | 0.60 | Thr | ACG | 6 | 0.09 |
| Gly | GGT | 42 | 1.15 | Thr | ACT | 70 | 1.06 |
| His | CAC | 14 | 0.40 | Trp | TGA | 106 | 1.83 |
| His | CAT | 56 | 1.60 | Trp | TGG | 10 | 0.17 |
| Ile | ATC | 90 | 0.22 | Tyr | TAC | 42 | 0.25 |
| Ile | ATT | 740 | 1.78 | Tyr | TAT | 296 | 1.75 |
| Leu | CTA | 60 | 0.43 | Val | GTA | 96 | 1.79 |
| Leu | CTC | 2 | 0.01 | Val | GTC | 0 | 0.01 |
| Leu | CTG | 4 | 0.03 | Val | GTG | 12 | 0.22 |
| Leu | CTT | 62 | 0.44 | Val | GTT | 106 | 1.98 |

**Table S10** The RSCU and codon statistics of *Parthenolecanium corni*. The asterisk represents termination codon.

| AA | Codon | Count | RSCU | AA | Codon | Count | RSCU |
| --- | --- | --- | --- | --- | --- | --- | --- |
| * | TAA | 11 | 1.91 | Leu | TTA | 290 | 5.01 |
| * | TAG | 0 | 0.09 | Leu | TTG | 19 | 0.33 |
| Ala | GCA | 11 | 2.00 | Lys | AAA | 201 | 1.90 |
| Ala | GCC | 0 | 0.09 | Lys | AAG | 11 | 0.10 |
| Ala | GCG | 0 | 0.09 | Met | ATA | 599 | 1.93 |
| Ala | GCT | 10 | 1.82 | Met | ATG | 22 | 0.07 |
| Arg | CGA | 12 | 2.00 | Phe | TTC | 24 | 0.11 |
| Arg | CGC | 0 | 0.08 | Phe | TTT | 409 | 1.89 |
| Arg | CGG | 0 | 0.08 | Pro | CCA | 43 | 2.51 |
| Arg | CGT | 11 | 1.83 | Pro | CCC | 3 | 0.18 |
| Asn | AAC | 56 | 0.37 | Pro | CCG | 0 | 0.03 |
| Asn | AAT | 246 | 1.63 | Pro | CCT | 22 | 1.29 |
| Asp | GAC | 3 | 0.13 | Ser | AGA | 47 | 1.45 |
| Asp | GAT | 44 | 1.87 | Ser | AGC | 1 | 0.03 |
| Cys | TGC | 3 | 0.43 | Ser | AGG | 1 | 0.03 |
| Cys | TGT | 11 | 1.57 | Ser | AGT | 31 | 0.96 |
| Gln | CAA | 30 | 1.88 | Ser | TCA | 106 | 3.27 |
| Gln | CAG | 2 | 0.13 | Ser | TCC | 2 | 0.06 |
| Glu | GAA | 50 | 1.85 | Ser | TCG | 1 | 0.03 |
| Glu | GAG | 4 | 0.15 | Ser | TCT | 70 | 2.16 |
| Gly | GGA | 42 | 2.42 | Thr | ACA | 92 | 3.05 |
| Gly | GGC | 0 | 0.03 | Thr | ACC | 4 | 0.13 |
| Gly | GGG | 5 | 0.29 | Thr | ACG | 0 | 0.02 |
| Gly | GGT | 22 | 1.27 | Thr | ACT | 24 | 0.80 |
| His | CAC | 3 | 0.16 | Trp | TGA | 50 | 1.85 |
| His | CAT | 34 | 1.84 | Trp | TGG | 4 | 0.15 |
| Ile | ATC | 28 | 0.14 | Tyr | TAC | 13 | 0.18 |
| Ile | ATT | 367 | 1.86 | Tyr | TAT | 132 | 1.82 |
| Leu | CTA | 23 | 0.40 | Val | GTA | 44 | 1.49 |
| Leu | CTC | 1 | 0.02 | Val | GTC | 0 | 0.02 |
| Leu | CTG | 1 | 0.02 | Val | GTG | 1 | 0.03 |
| Leu | CTT | 13 | 0.23 | Val | GTT | 73 | 2.46 |

**Table S11** Detailed information of alignment and corresponding optimal tree for three datasets. Partition scheme (t), where the number in parentheses indicates the number of partitions.

| Datasets | Partition scheme (t) | Model | Parameters (k) | ln(Lik) | AIC | BIC | BS>=95 | BS<95 | Saturation | SNR | PIS |
| --- | --- | --- | --- | --- | --- | --- | --- | --- | --- | --- | --- |
| AA | MP(4) | - | 427 | -386285.3815 | 773424.7630 | 776098.4348 | 117 | 30 | 0.8784 | 1.7063 | 3409 |
| AA | NP(1) | mtInv+F+C60 | 377 | -387430.1695 | 775614.3390 | 777974.9345 | 117 | 30 | 0.8779 | 1.6949 | 3409 |
| AA | FP(13) | - | 637 | -386637.3580 | 774548.7160 | 778537.3083 | 116 | 31 | 0.8784 | 1.7077 | 3409 |
| AA | NP(1) | MFP | 331 | -387957.3433 | 776576.6866 | 778649.2519 | 119 | 28 | 0.8820 | 1.6857 | 3409 |
| P123R | FP(15) | - | 598 | -834866.6265 | 1670929.2530 | 1675459.1760 | 125 | 22 | 0.5803 | 1.5502 | 13012 |
| P123R | MP(8) | - | 446 | -835720.4346 | 1672332.8690 | 1675711.3740 | 126 | 21 | 0.5807 | 1.5515 | 13012 |
| P123R | NP(1) | MFP | 322 | -847220.2609 | 1695084.5220 | 1697523.7110 | 123 | 24 | 0.5782 | 1.5285 | 13012 |
| P12R | MP(8) | - | 431 | -475008.3335 | 950878.6670 | 954008.5819 | 115 | 32 | 0.4861 | 1.5023 | 9216 |
| P12R | FP(15) | - | 566 | -474587.7551 | 950307.5102 | 954417.7929 | 117 | 30 | 0.4858 | 1.5004 | 9216 |
| P12R | NP(1) | MFP | 320 | -480213.5389 | 961067.0778 | 963390.9126 | 118 | 29 | 0.4838 | 1.4770 | 9216 |
